# PNUTS/PP1 Regulates RNAPII-Mediated Gene Expression and Is Necessary for Developmental Growth

**DOI:** 10.1371/journal.pgen.1003885

**Published:** 2013-10-31

**Authors:** Anita Ciurciu, Louise Duncalf, Vincent Jonchere, Nick Lansdale, Olga Vasieva, Peter Glenday, Andreii Rudenko, Emese Vissi, Neville Cobbe, Luke Alphey, Daimark Bennett

**Affiliations:** 1 Institute of Integrative Biology, University of Liverpool, Liverpool, United Kingdom; 2 Department of Zoology, Oxford University, Oxford, United Kingdom; Berlin Institute for Medical Systems Biology, Germany

## Abstract

In multicellular organisms, tight regulation of gene expression ensures appropriate tissue and organismal growth throughout development. Reversible phosphorylation of the RNA Polymerase II (RNAPII) C-terminal domain (CTD) is critical for the regulation of gene expression states, but how phosphorylation is actively modified in a developmental context remains poorly understood. Protein phosphatase 1 (PP1) is one of several enzymes that has been reported to dephosphorylate the RNAPII CTD. However, PP1's contribution to transcriptional regulation during animal development and the mechanisms by which its activity is targeted to RNAPII have not been fully elucidated. Here we show that the *Drosophila* orthologue of the PP1 Nuclear Targeting Subunit (dPNUTS) is essential for organismal development and is cell autonomously required for growth of developing tissues. The function of dPNUTS in tissue development depends on its binding to PP1, which we show is targeted by dPNUTS to RNAPII at many active sites of transcription on chromosomes. Loss of dPNUTS function or specific disruption of its ability to bind PP1 results in hyperphosphorylation of the RNAPII CTD in whole animal extracts and on chromosomes. Consistent with dPNUTS being a global transcriptional regulator, we find that loss of *dPNUTS* function affects the expression of the majority of genes in developing 1^st^ instar larvae, including those that promote proliferative growth. Together, these findings shed light on the *in vivo* role of the PNUTS-PP1 holoenzyme and its contribution to the control of gene expression during early *Drosophila* development.

## Author Summary

During development, cells rely on appropriate patterns of gene expression to regulate metabolism in order to meet cellular demands and maintain rapid tissue growth. Conversely, dysregulation of gene expression is critical in various disease states, such as cancer, and during ageing. A key mechanism that is ubiquitously employed to control gene expression is reversible phosphorylation, a molecular switch that is used to regulate the activity of the transcriptional machinery. Here we identify an enzyme that binds to and regulates the phosphorylation state of RNA Polymerase II, a central component of the general transcription machinery. We also show that an essential role of this enzyme is to support normal patterns of gene expression that facilitate organismal growth. These findings are not only of relevance to the understanding of normal enzyme function but may also assist in the development of therapeutic strategies for the treatment of aberrant patterns of gene expression that occur during ageing and disease progression.

## Introduction

Development must be tightly coupled with cellular metabolism to ensure that necessary nutritional and energetic requirements are met and the available resources are utilised effectively to sustain appropriate levels of tissue growth. A particularly dramatic example of how development is coupled to metabolism is during the transition through the larval stages of *Drosophila* development, during which animals accumulate a 200-fold increase in body mass. The metabolic needs to sustain this rapid expansion are underpinned by transcriptional programmes initiated in the embryo; as maternal products become exhausted, large numbers of zygotically expressed genes, responsible for converting raw materials into cell mass, are induced to sustain developmental growth [1]. Elucidating what factors are necessary to drive these transcriptional programmes is not only critical for understanding tissue and organism size regulation during normal development, but is also important for understanding numerous disease processes characterized by inappropriate gene expression.

Reversible phosphorylation plays important roles in the regulation of transcriptional networks and in coordinating spatial and temporal patterns of gene expression. Phosphorylation of RNA polymerase II (RNAPII) at multiple sites on its C-terminal domain (CTD) is critical for gene expression and its regulation [2]. Different phospho-forms of the CTD appear at different stages of the transcription cycle, and these are thought to facilitate initiation, elongation and termination by recruiting specific histone and RNA modifiers [3], [4]. The consensus view from studies of RNAPII occupancy in budding yeast is that there is a stereotypical pattern of phosphorylation at most gene loci during the transcription cycle [5], [6]. However, numerous lines of evidence suggest that there is active control of CTD phosphorylation in response to environmental cues [7]–[9] and during developmental transitions, e.g. in which restriction of CTD phosphorylation to particular lineages [10] is used to control cell fate [11]. Furthermore, studies of the enzymes responsible for regulating CTD phosphorylation indicate that phosphorylation may be modified at specific loci to determine gene-specific patterns of expression [12], [13].

Serine/threonine protein phosphatase type 1 (PP1) is one of four protein phosphatases known to contribute to the regulation of CTD phosphorylation [14], the others being FCP1 [15], SCP1 [11] and Ssu72 [16]. In *Drosophila*, PP1 is found at multiple sites on chromosomes where it has been postulated to play important roles in regulating developmentally controlled gene expression [17], [18]. However, analysing the role of PP1 in transcriptional regulation has been complicated by its pleiotropic roles [19] and broad *in vitro* substrate specificity. *In vivo*, PP1 has been shown to associate with different targeting subunits that restrict its activity towards particular substrates [20]. Therefore, a full understanding of PP1 function requires the identification and characterisation of these regulatory proteins.

In mammalian cells, the PP1 Nuclear Targeting Subunit (PNUTS) is one of the two most abundant PP1-interacting proteins in the nucleus [21] and is known to be chromatin-associated during interphase and not during mitosis [22], [23]. Its reassociation with chromatin during telophase and its ability to augment chromosome decondensation *in vitro* [24] and *in vivo* [25] have indicated a possible role in cell cycle progression. Several lines of evidence also indicate that PNUTS is required for cell survival [26]–[30] and contributes to cellular responses to environmental stress, including hypoxia [31] and DNA damage [32]. These roles may be especially important during ageing since loss of PNUTS expression is associated with an age-dependent increase in cardiomyocyte apoptosis and decline in cardiac function [33]. Targeting of PNUTS to chromatin is likely to be in part through association with the DNA-binding factor Tox4/Lcp1 [25], [34], which is capable of recognising DNA adducts generated by platinum anticancer drugs [35]. PNUTS and Tox4 have also been reported to form a stable multimeric complex with Wdr82 [25], which was previously identified as an integral component of a distinct complex containing Set histone H3-Lys4 methyltransferases. Although the role of Wdr82 bound to PNUTS is not known, Wdr82 may mediate interactions with initiating and early elongating RNAPII by recognising Ser5-phosphorylated CTD, as it does when it is associated with the Set1 complex [36]. A role for PNUTS in transcription has been further suggested by recent reports that it associates with RNAPII complexes [37]. Despite these insights, an understanding of the physiological roles of PNUTS remains incomplete.

Here we show that null mutants in the *D. melanogaster* orthologue of PNUTS (*dPNUTS*), display a larval growth defect and are larval lethal. Mutant clones show a cell autonomous growth defect and are eliminated from wild type epithelia due to cell competition. RNA-Sequencing (RNA-Seq) analysis indicates that *dPNUTS* affects the expression of the majority of genes in 1^st^ instar larvae, including those that are highly expressed and are involved in cellular metabolism and larval development. The function of *dPNUTS* in tissue development is dependent on binding to the catalytic subunit of Protein phosphatase 1 (PP1), which is targeted by dPNUTS to RNA polymerase II in cell extracts and at many active sites of transcription on polytene chromosomes. Loss of *dPNUTS* function, or displacement of dPNUTS-PP1 using a non-PP1 binding mutant of *dPNUTS*, results in hyperphosphorylation of the C-terminal domain of RNA Polymerase II in whole animal extracts and on chromosomes. Taken together, these data suggest that dPNUTS-PP1 is a global regulator of gene expression via effects on RNAPII phosphorylation and is required in larvae to promote normal developmental growth.

## Results

### PNUTS is highly conserved across metazoa

Sequence homology searches have suggested that PNUTS is a metazoan PP1-binding protein [38]. However, its absence from species such as *C. elegans* indicates that it has not been retained in all metazoa. *D. melanogaster* contains one gene encoding PNUTS: *CG33526/dPNUTS*. Comparison of full-length PNUTS cDNA and genomic sequences shows that all four of d*PNUTS* intron/exon boundaries are shared with human *PNUTS* (hPNUTS; Figure S1), indicating that h*PNUTS* and d*PNUTS* are derived from a single ancestral gene. dPNUTS encodes two protein isoforms: dPNUTS and dPNUTS-S, a truncated version containing only the N-terminal region of dPNUTS. There is extensive homology between dPNUTS and mammalian PNUTS in a number of protein domains (Figure S1).

### dPNUTS is expressed in developing tissues and localises to transcriptionally active sites on interphase chromosomes

**Figure 1:**
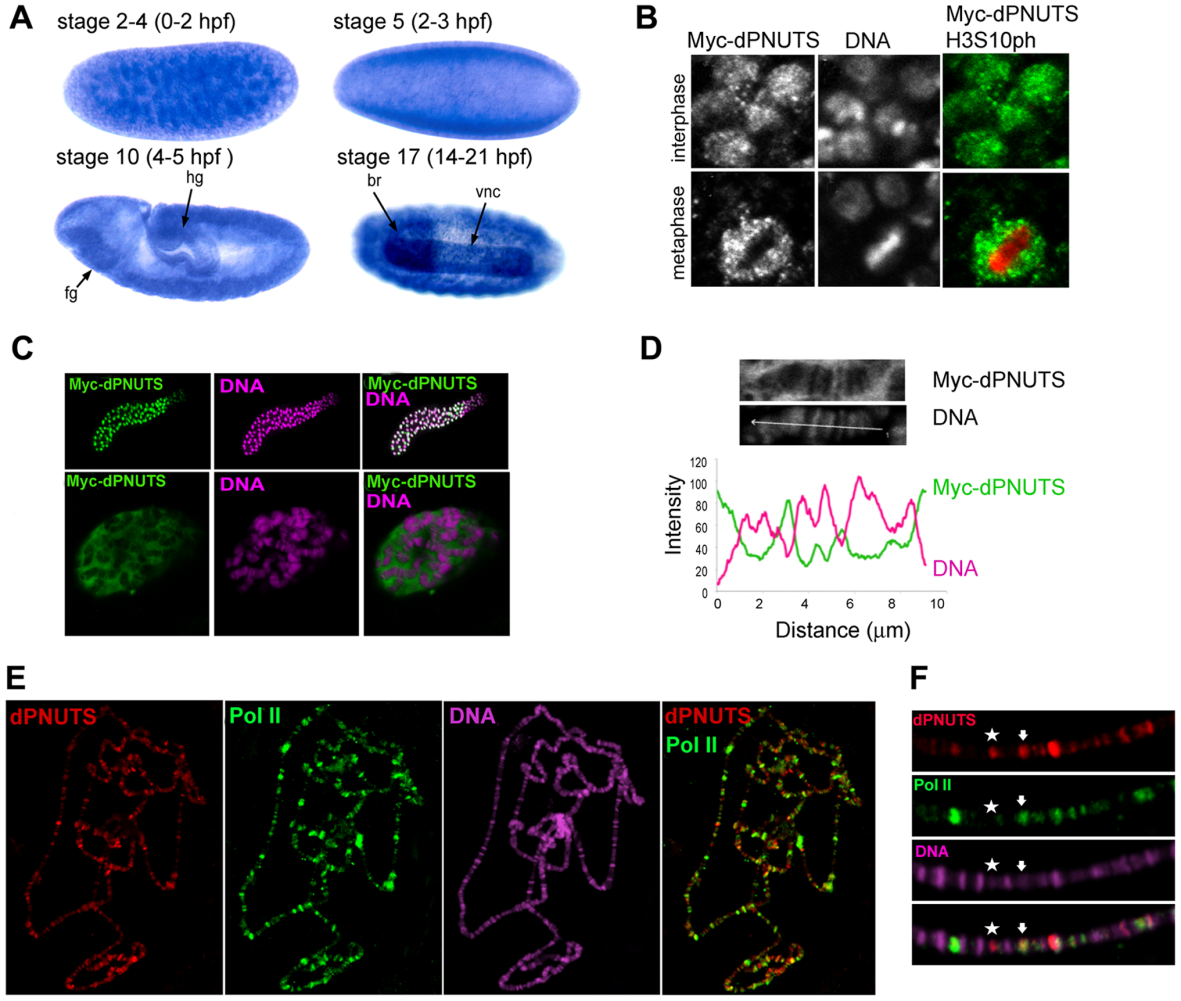
dPNUTS is a nuclear protein that colocalises with transcriptionally active RNAPII on salivary gland polytene chromosomes. A) Distribution of *dPNUTS* transcripts detected by RNA *in situ* hybridization; *dPNUTS* transcripts are maternally provided (top left) and are ubiquitously distributed in embryos at cellularisation (top right). At gastrulation, d*PNUTS* mRNA levels are enriched in the germband and in the fore- and hind-gut (fg and hg, respectively). Later, d*PNUTS* is highly expressed in the brain (br) and ventral nerve cord (vnc). Embryonic stage and approximate age, hours post fertilization (hpf), are indicated. B) 3^rd^ instar wing discs stained to reveal the distribution of ectopically expressed Myc-tagged dPNUTS (green in merge), Histone H3S10ph (red in merge, marking mitotic nuclei) and DNA. C) Images of whole mount salivary gland and magnified images of an individual nucleus (below), stained to show the localization of Myc-tagged dPNUTS (green in merge) and DNA (magenta in merge). D) Line scans of images in C) reveal that Myc-tagged dPNUTS is localised to interbands that stain weakly for DNA. Fluorescence intensity of anti-Myc antibody and TOPRO-3 staining was measured along a line through the indicated chromosomal region in the images shown. The profile plot below shows that the peaks of Myc-PNUTS and DNA of staining do not overlap. E) Polytene chromosomes from salivary gland squashes showing that dPNUTS localises to a number of discrete bands that are broadly distributed. F) Merging of the green signal representing dPNUTS with the red signal representing RNAPII Ser2-P (H5) identifies sites where these two proteins co-localize (example indicated with arrow). The relative signals of dPNUTS and RNAPII Ser2-P vary between sites, but the majority dPNUTS loci colocalize with RNAPII Ser2-P staining (star indicates example where only dPNUTS staining is visible).

PNUTS has been identified in all mammalian tissues so far examined [22], [23], but the highest level of expression is reported to be in testis, brain, and intestine. *In situ* hybridisation revealed that *dPNUTS* transcripts are maternally provided and are uniformly distributed in most tissues during *Drosophila* embryogenesis. However, strikingly, there was stronger staining in the developing gut and in the nervous system during phases of rapid development (Figure 1A). To determine the subcellular distribution of dPNUTS, we generated transgenic fly lines capable of expressing epitope-tagged dPNUTS under UAS-GAL4 control. Ectopic dPNUTS shows a similar subcellular localisation to mammalian PNUTS: dPNUTS is nuclear and associates with chromatin during interphase when ectopically expressed in the wing disc, but is excluded from condensed chromosomes at metaphase (Figure 1B). In polytene nuclei, ectopic dPNUTS was visible in both the nucleoplasm and on polytene chromosomes as revealed by co-staining the DNA with Hoechst (Figure 1C,D). Strong Hoechst staining is associated with condensed chromosomal bands, which contain a high concentration of DNA, whilst weak, or no Hoechst signal is detected in interband regions of less tightly packed chromatin, which are thought to contain actively transcribed genes. dPNUTS is predominantly associated with regions of less condensed DNA corresponding to interbands that stain weakly with Hoechst (Figure 1D).

To examine the chromosomal association of dPNUTS further, we generated antibodies specific to dPNUTS and used them to stain polytene chromosomes from 3^rd^ instar larval salivary glands. Although the dPNUTS antibodies worked well on polytene squash preparations we were unable to obtain a reliable signal from whole tissue mount preparations. We found that dPNUTS is localised at a large number of discrete sites of varying strength along all the chromosomes. To confirm the specificity of the dPNUTS antiserum on polytene squashes, we knocked down *dPNUTS* levels in salivary glands using heritable double-stranded RNA interference (RNAi). Flies carrying an inverted repeat (^IR^) construct under UAS control were crossed to a salivary gland GAL4 source to induce expression of intron-spliced hairpin dsRNA for *dPNUTS* in the progeny. In squash preparations from relatively normal looking glands expressing *UAS-dPNUTS^IR^* we found greatly reduced dPNUTS staining (Figure S2). To explore the possibility that dPNUTS may be associated with transcriptionally active sites, we performed double labelling experiments with antibodies against the active form of RNA polymerase II (RNAPII). We found that the relative levels of dPNUTS and RNAPII vary at many sites, but, on close inspection, it is clear that the dPNUTS antibody marks a large number of transcriptionally active sites containing active RNAPII (H5 antibody, detecting RNAPII Ser2-P) (Figure 1E, F), suggesting that *dPNUTS* might have a role in transcriptional regulation.

### *dPNUTS* loss of function results in larval growth arrest and defective tissue development

**Figure 2:**
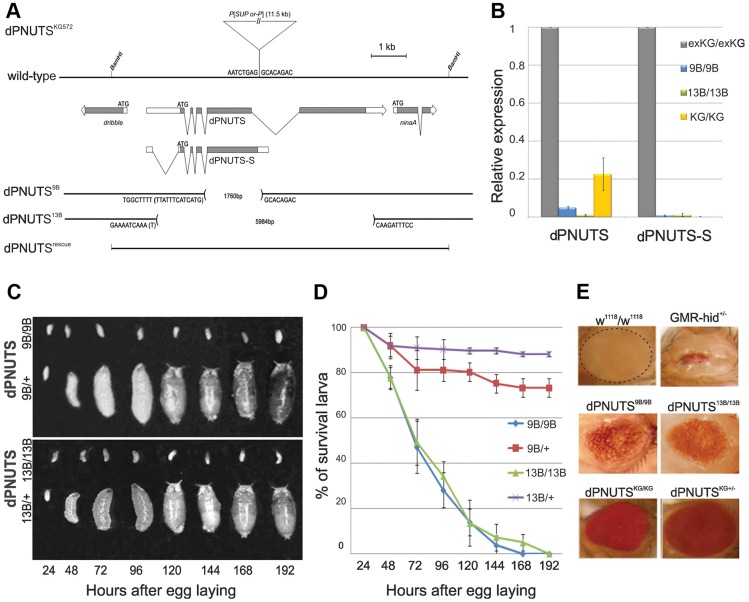
*dPNUTS* loss of function results in larval growth arrest and defective tissue development. A) Genomic region showing *dPNUTS* locus flanked by *dribble* and *ninaA*. Coding regions of the genes is represented by shading. *dPNUTS* produces two transcripts *dPNUTS* and *dPNUTS-S*. *ninaA* is a non-essential gene that is expressed solely in the eye to regulate rhodopsin synthesis [75], [76]. *dPNUTS^KG572^* contains a *P* element insertion in an untranslated region of *dPNUTS*. The extent of deletions in *dPNUTS^9B^* and *dPNUTS^13B^* resulting from imprecise excision of this element is indicated, together with the genomic sequence of the breakpoints. The *dPNUTS* genomic rescue construct, which contains the coding region of *ninaA*, and the 5′ end of *dribble*, is indicated. B) Levels of *dPNUTS* and *dPNUTS-S* transcripts produced in homozygous *dPNUTS^exKG^*, *dPNUTS^KG572^*, *dPNUTS^9B^* and *dPNUTS^13B^* larvae, as determined by qRT-PCR. *dPNUTSe^exKG^* is a revertant strain in which the *P* element had precisely excised. C) Images of homozygous mutant and control (heterozygous sibling) larvae at different time points after egg laying as indicated. D) Graph showing percentage of surviving larvae over time for each genotype, as indicated. E) Images of adult female eyes. Homozygous *dPNUTS* mutant eyes are smaller than controls (isogenic *w^1118^* strain), but are able to form some facets, unlike eyes expressing the proapototic gene *hid* under *GMR* control.

To determine the *in vivo* role of *Drosophila PNUTS*, we generated two deletion alleles, *dPNUTS^9B^* and *dPNUTS^13B^*, by imprecise excision of a *P* element transposon (*P[SUPor-P] dPNUTS^KG00572^*, referred to as *dPNUTS^KG572^* hereafter). Molecular analysis revealed that virtually all of the fourth coding exon of *dPNUTS* is deleted in *dPNUTS^9B^*, and the entire coding region, including the translation start site, is deleted in *dPNUTS^13B^* (Figure 2A). Consistent with these findings, quantitative RT-PCR analyses revealed the absence or almost complete loss of *dPNUTS* transcripts in *dPNUTS^9B^* and *dPNUTS^13B^* homozygotes. *dPNUTS* levels were also greatly reduced in *dPNUTS^KG572^* homozygotes compared to revertant controls (*dPNUTS^exKG^*) in which the *P* element had been precisely excised (Figure 2B). *dPNUTS^9B^* and *dPNUTS^13B^* are recessive lethal in combination with each other and over *Df(2L)ast4*, a deficiency that removes the *dPNUTS* gene. The phenotype of *dPNUTS^9B^* and *dPNUTS^13B^* homozygotes was indistinguishable from that of *dPNUTS^9B^* or *dPNUTS^13B^*/*Df(2L)ast4* hemizygotes, so we conclude that the excision alleles have little or no residual *dPNUTS* function. To confirm that disruption of the *dPNUTS* transcription unit is responsible for the larval lethality, we generated transgenic flies carrying a genomic fragment containing the entire *PNUTS* locus. A single copy of the transgenic construct was capable of fully rescuing the homozygous lethality of *dPNUTS^9B^* and *dPNUTS^13B^* mutants (Table S1).

To examine the lethal phase of *dPNUTS* mutants we combined the mutant alleles with a GFP-balancer chromosome and examined the development of mutant (non-GFP) larvae alongside their heterozygous (GFP marked) siblings. Homozygous *dPNUTS^9B^* and *dPNUTS^13B^* animals developed to 1^st^ instar larvae but died in the ensuing 8 days without further growth and development (Figure 2C, D). To further assess the requirement for *dPNUTS* in tissue development we made use of the *ey-FLP* system to produce genetically mosaic flies that are otherwise heterozygous but in which the eye is composed exclusively of cells homozygous mutant for *dPNUTS*. Cells that are not derived from the homozygous mutant cells are eliminated by eye-specific expression of the pro-apoptotic gene *hid* [39]. Eyes of heterozygous *dPNUTS^KG572^*, *dPNUTS^9B^* and *dPNUTS^13B^* flies resembled wild type. Flies with eyes homozygous for *dPNUTS^KG572^* were modestly reduced in size, with fewer and poorly organised ommatidia. Eyes homozygous for either *dPNUTS^9B^* or *dPNUTS^13B^* showed a more severe effect, indicating that cells lacking *dPNUTS* are incapable of developing into adult eyes (Figure 2E).

### *dPNUTS* mutant cells fail to compete with wild type cells and are removed from developing epithelia

**Figure 3:**
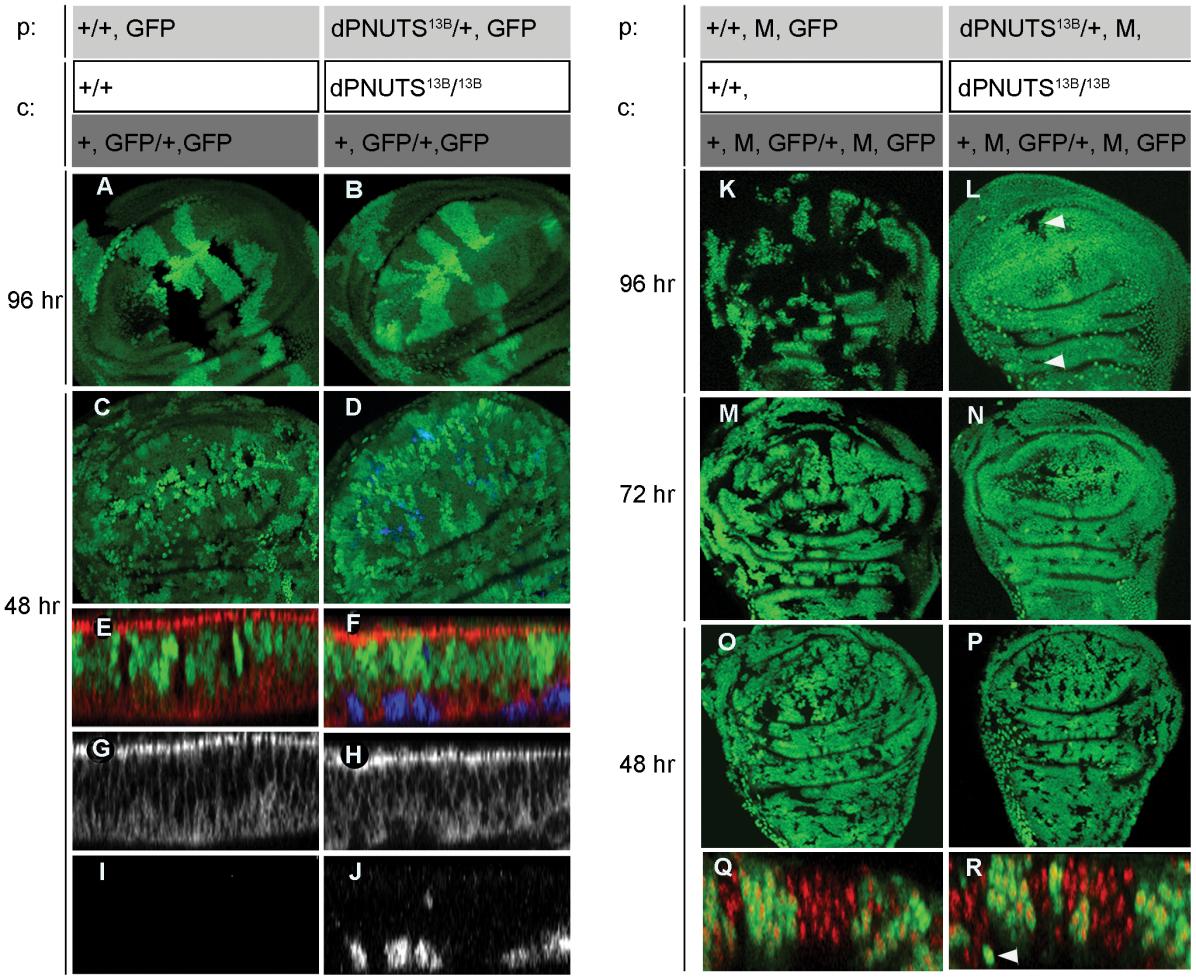
*dPNUTS* mutant clones reveal a cell autonomous growth defect in developing tissues. Clones (marked by absence of GFP) of either wild-type or *dPNUTS^13B^* mutant cells are shown in wing imaginal discs obtained from 3^rd^ instar larvae. Clones were induced in a wild type (A–J) or *Minute* (*M*) mutant background (K–R) 48 hr, 72 hr or 96 hr prior to dissection, as indicated. The parental (p) genotypes are indicated, along with the genotype of clones (c) generated by FLP-mediated mitotic recombination and are coded with grayscale to indicate the relative level of GFP expression. A–J, wing discs were stained for activated caspase shown in blue, and in cross sections (E–J), apico-lateral junctions are marked by discs-large staining in red; GFP is shown in green. In cross sections Q–R, DNA is shown in red. Arrowheads in panel L indicate the presence of *dPNUTS^13B^* GFP-negative clones in a *Minute* (*M*) mutant background. +, *M*, GFP/+, *M*, GFP twinspot clones, indicated by arrowhead in panel R, were almost never observed because of a severe growth defect.

To understand more about the cellular role of *dPNUTS*, we generated clones of homozygous null *dPNUTS* mutant cells in otherwise *dPNUTS* heterozygous wing imaginal discs during early or mid-larval development using *Flp*/*FRT*-mediated recombination [40] and analysed them at the wandering 3^rd^ instar larval stage. To do this we used a heat shock inducible Flipase (Flp) enzyme to induce mitotic recombination between two FRT chromatids, one of which carried a mutant *dPNUTS* allele and the other which expressed a GFP marker. Mitotic recombination events produce a GFP-negative cell clone that are homozygous for the mutant allele, together with a “twin-spot” marked by the presence of two copies of GFP. Surrounding heterozygous tissue is labelled with one copy of GFP. We failed to recover homozygous mutant cells when clones were induced in early 1^st^ instar larvae, whereas wild type cells induced at the same stage proliferated to generate large clonal patches (Figure 3A, B). When we shortened the time between clone induction and analysis by inducing clones later on in 2^nd^ instar larvae, we were able to observe very small patches of *dPNUTS* mutant cells (Figure 3C, D). However, in optical cross sections through the tissue it was apparent that mutant cells accumulated at the basal face of the epithelium and stained positive for cleaved caspase antibody (Figure 3E–J), indicating that *dPNUTS* mutant cells were undergoing cell death. This prompted us to examine whether clones were dying due to cell competition, a process in which slow-growing cells are eliminated by their faster-growing neighbours. To test this, we gave the *dPNUTS* mutant cells a growth advantage by generating them in tissues that were heterozygous for a dominant *Minute* (*M*) allele of *RpL27A*. Notably, under conditions in which *dPNUTS* mutant clones in a wild-type background are normally eliminated, we recovered *dPNUTS* clones in *M*/*+* discs (Figure 3K, L and Figure S3) and mutant clones spanned the entire wing disc epithelium indicating they were not being eliminated (Figure 3Q, R and Figure S3). However, mutant clones colonised a significantly smaller area of *M*/*+* discs compared with wild-type clones, indicating that they were still growth impaired (compare Figure 3K and 3L).

### *dPNUTS* mutants deregulate the expression of the majority of genes in 1^st^ instar larvae

**Figure 4:**
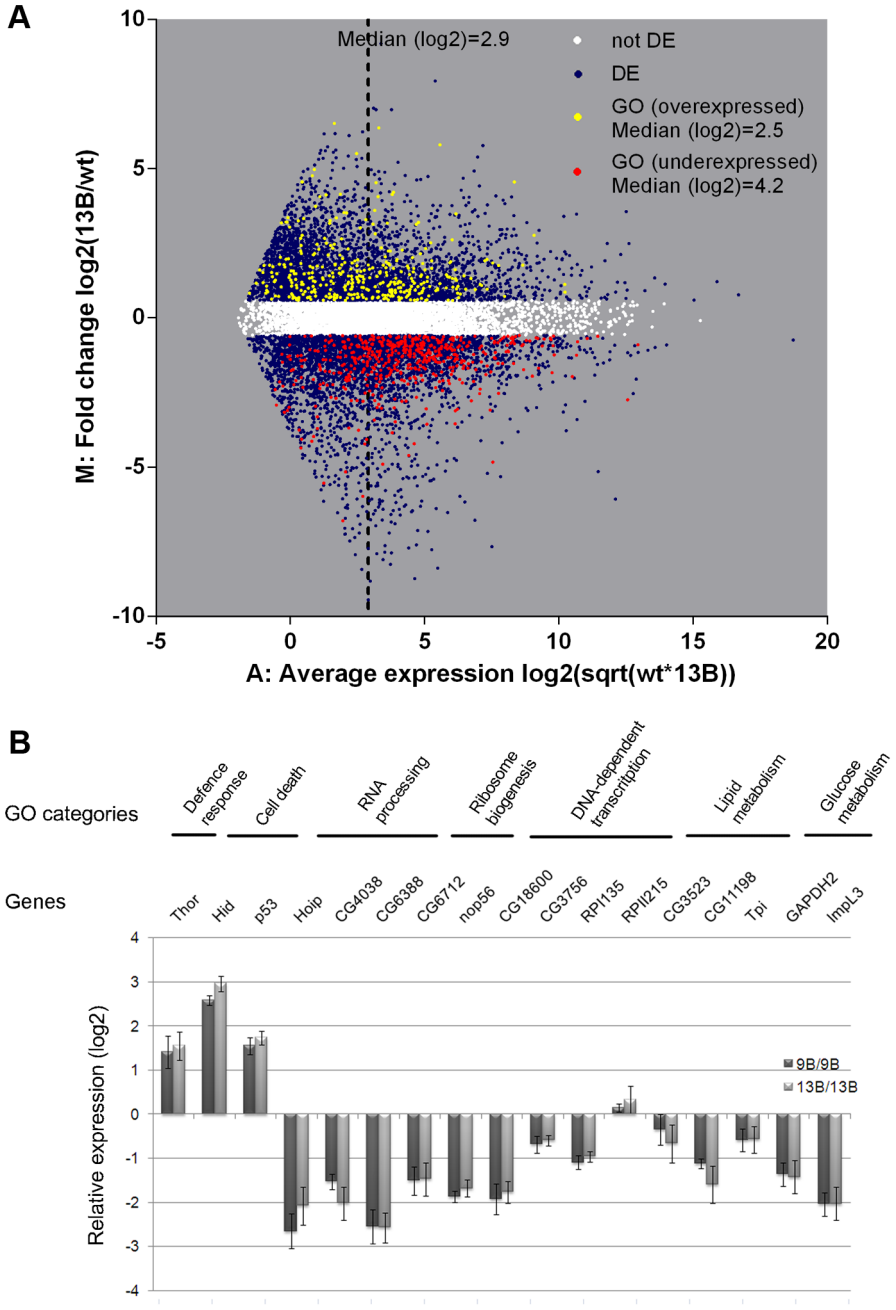
*dPNUTS* mutants deregulate the expression of the majority of genes in 1^st^ instar larvae, including highly expressed genes involved in cellular metabolism and proliferative growth. A) MA plot of RNA-Seq data in which the log_2_ of the ratio of abundance of each transcript between *dPNUTS^13B^* (13B) mutant and *w^1118^* (WT) control (M) is plotted against the log_2_ geometric average of abundance (FPKM) in both conditions (A). Transcripts with an FPKM of less than 0.25, which are a source of noise in these plots, are not shown for clarity. Transcripts that are differentially expressed (DE) by <0.67 or >1.5 fold are shown in grey; unaffected transcripts are shown in white. Loci corresponding to enriched Gene Ontology (GO) terms amongst the differentially expressed genes relative to the entire genome are highlighted in yellow (enriched amongst overexpressed genes) or red (enriched amongst underexpressed genes). Log_2_ median expression for genes expressed in WT and 13B is indicated with a dashed line. Log_2_ median expression for genes belonging to GO categories is given in the legend. A complete list of GO categories is provided in the Supplementary information. B) Expression levels of the indicated genes in *dPNUTS^9B^*/*dPNUTS^9B^* and *dPNUTS^13B^*/*dPNUTS^13B^* mutant larvae, relative to control (*w^1118^*) larvae, determined by qRT-PCR. Error bars represent the SE (n≥3 biological replicates). The GO categories to which the genes belong are shown at the top.

To obtain an insight into the molecular basis for the growth defects in *dPNUTS* mutants and assess the impact of *dPNUTS* loss of function on gene expression, we analysed the transcriptomic signature of *dPNUTS^9B^* and *dPNUTS^13B^* mutant larvae by RNA-Seq. The control for these experiments was an isogenic strain that carried the same background mutation (w*^1118^*) as the *dPNUTS* mutant strains. Homozygous *dPNUTS^9B^* and *dPNUTS^13B^* mutant 1^st^ instar larvae had widespread changes in gene expression compared to control animals of the same stage (Figure 4A), with a comparable pattern of genes being affected in both mutants (Figure S4). In total, approximately 30% of genes (2819/9483) previously reported to be expressed in 1^st^ instar larvae [40] were underexpressed, and a similar proportion (2850/9483) were overexpressed >1.5-fold in both dPNUTS^9B^ and *dPNUTS^13B^* mutant animals relative to control larvae. Therefore, we conclude that disruption of *dPNUTS* function affects the expression of the majority of genes in developing 1^st^ instar larvae.

To assess whether there was any enrichment of genes belonging to functionally-related biological processes, we analysed the distribution of Gene Ontology (GO) terms amongst differentially expressed genes. When compared to the frequency of GO terms amongst all genes encoded by the genome, we observed significant (*P*≤10^−4^) enrichment of terms for cell death and stress responses amongst genes overexpressed in *dPNUTS* mutants (Figure S5, Table S2). Overexpression of these groups of genes might indicate that the animals are under stress and is consistent with their poor survival. The most significantly enriched GO terms amongst the underexpressed genes in *dPNUTS* mutants, were terms for cellular metabolic processes that drive proliferative growth, including ribosome biogenesis, rRNA processing, translation and metabolism of energy sources (Figure S5, Table S2). We observed a similar pattern of GO enrichment when comparing differentially expressed genes in the *dPNUTS* mutants to genes expressed in our developmentally matched control (Table S3). These patterns of transcriptional change are consistent with the larval growth defect exhibited by the *dPNUTS* mutants. In addition, Ingenuity analysis identified a number of different transcriptional networks involved in organismal growth that are likely to be affected by loss of *dPNUTS* (Table S4).

While these analyses provide biological insight into the likely processes underpinning the *dPNUTS* mutant phenotype, it is important to note that the enrichment of biologically-relevant GO categories is correlated with the expression level of the representative genes in 1^st^ instar larvae (Figure 4A). Indeed, GO categories pertaining to cellular metabolism are also enriched amongst highly expressed genes in the control (median expression level >(log_2_)2.9 FPKM; data not shown). Taken together with the widespread effects on transcript abundance, these data indicate that *dPNUTS* globally affects gene expression and in 1^st^ instar larvae is required to promote expression of highly expressed genes that support developmental growth.

To confirm the RNA-Seq results, we selected genes representative of enriched GO categories for quantitative real-time qRT-PCR analysis. Measurements of relative mRNA expression level determined by qRT-PCR were consistent with our RNA-Seq data (Figure 4B, Table S5).

### dPNUTS binds to and colocalises with PP1 on chromosomes

**Figure 5:**
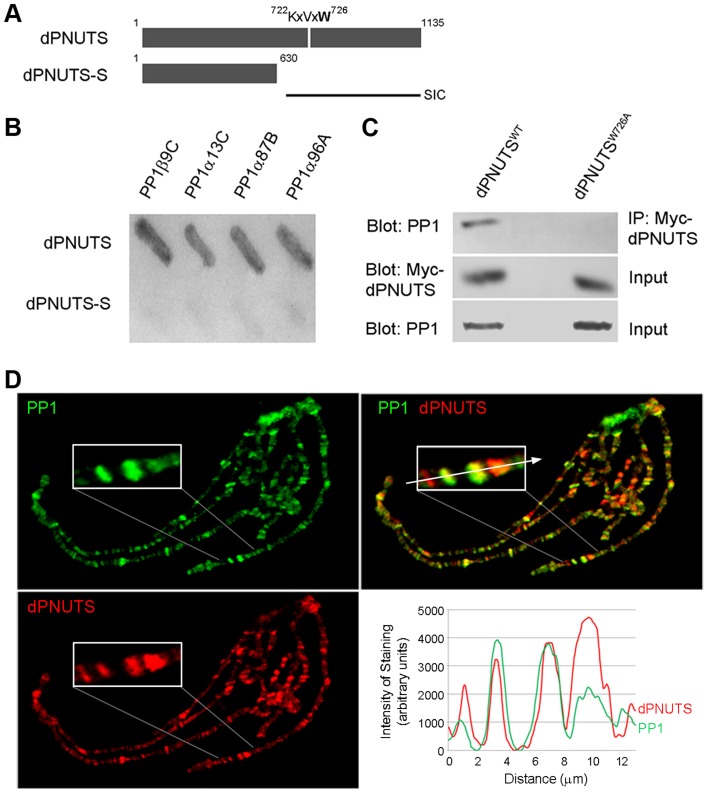
dPNUTS binds to and co-localises with PP1 on chromosomes. A) Predicted domain structure of dPNUTS and dPNUTS-S proteins, indicating the position of the putative PP1-binding motif (residues 722–726), which is located in the shortest yeast two-hybrid interacting clone (SIC) of dPNUTS. B) Beta-galactosidase assays showing binding of dPNUTS but not dPNUTS-S to all four *D. melanogaster* PP1 isoforms in the yeast two-hybrid system. C) dPNUTS^WT^, but not dPNUTS^W726A^, co-precipitates PP1 from nuclear extracts from adult flies. *da-GAL4 UAS-HM-dPNUTS^WT^* and *da-GAL4 UAS-HM-dPNUTS^W726A^* fly extracts were subjected to immunoprecipitation (IP) with Myc antibodies followed by immunoblotting with PP1 antibodies. Blots of total lysates confirmed levels of HM-tagged dPNUTS and PP1. D) dPNUTS and PP1 colocalise at many sites on polytene chromosomes. Inset is an enlarged view of the end of the X chromosome where this is clearly visible. Plot of fluorescence intensity of anti-PP1 and dPNUTS antibody staining, measured along a line through the indicated chromosomal region, reveal the degree of colocalisation between PP1 and dPNUTS.

dPNUTS was originally isolated from a two-hybrid screen for putative PP1-binding proteins and contains a canonical PP1-binding motif - K/R, (x), V/I/L, x, F/W that in PNUTS/p99 is necessary for binding to, and inhibition of, PP1 [23], [41]. This motif (residues 722–726) is also contained within all the *dPNUTS* two-hybrid clones, including the shortest interacting fragment encoding residues 608 to 1135 [42], (Figure 5A). When we retested binding in the two-hybrid system with full-length proteins, dPNUTS, but not dPNUTS-S, interacted strongly with all four *D. melanogaster* PP1 isoforms (Figure 5B), consistent with a role for this motif in binding PP1. To determine the importance of the putative PP1-binding motif for interaction with PP1, we compared binding of endogenous PP1, to ectopically expressed wild type dPNUTS (dPNUTS^WT^) and a mutant form in which Trp726 was replaced with Ala (dPNUTS^W726A^). Immunoprecipitation with antibodies against Myc-tagged dPNUTS, followed by immunoblotting with antibodies against PP1, showed that PP1 co-precipitated very efficiently with dPNUTS^WT^ but not dPNUTS^W726A^ (Figure 5C), indicating that Trp726 is crucial for interaction with PP1.

To further explore the association between PP1 and dPNUTS *in vivo*, we examined the distribution of dPNUTS and PP1 on polytene chromosomes from 3^rd^ instar larvae. We previously reported that ectopic HA-tagged PP187B, the major PP1 isoform in *Drosophila* [43], localised to many discrete chromosomal loci [17], [18]. Like the ectopic protein, we found a large number of discrete sites widely dispersed along the chromosomes that were stained with an anti-peptide antibody to *Drosophila* PP1 (Figure 5D). When we co-stained for dPNUTS, we found that most sites staining for dPNUTS also stained strongly for PP1 although the relative staining varied greatly (Figure 5D).

### dPNUTS recruits PP1 to chromosomes

**Figure 6:**
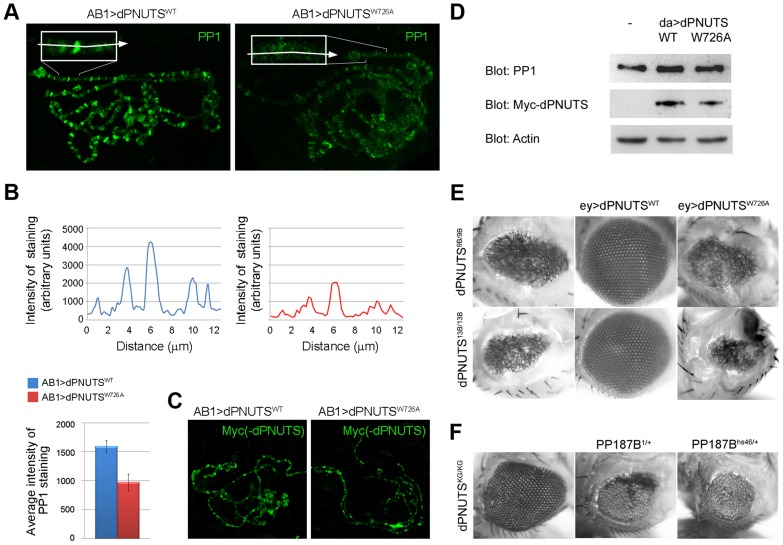
PP1 localisation is regulated by dPNUTS and binding to PP1 is important for dPNUTS function. A) Images of polytene chromosome squashes from salivary glands expressing either *dPNUTS^WT^* or *dPNUTS^W726A^* stained with PP1 (in green). Inset are enlarged views of the distal end of the X chromosome. Arrows indicate approximate lines along which quantitation of fluorescence (see B) was performed. B) Plots of line scans through the chromosomal region indicated in A, showing levels of PP1 staining in salivary glands expressing either *dPNUTS^WT^* or *dPNUTS^W726A^*. Bar graphs represent the average fluorescence in this region from 6 independent images/genotype. Genotypes are indicated by the colour key. C) Levels and distribution of Myc-dPNUTS on polytene chromosome squashes from salivary glands expressing either *dPNUTS^WT^* or *dPNUTS^W726A^*, as revealed by anti-Myc staining (green). D) Western blots showing levels of PP1 and Myc-dPNUTS, relative to Actin, in extracts from animals ectopically expressing *dPNUTS^WT^* or *dPNUTS^W726A^* under the control of *da-GAL4* (da>*dPNUTS^WT^* and da>*dPNUTS^W726A^*, respectively) compared to *w^1118^* control (−). E) Images of adult female eyes showing that the severely reduced eye phenotype of homozygous *dPNUTS* mutant eyes is fully rescued by ectopic expression of *dPNUTS^WT^* but not *dPNUTS^W726A^*. F) Homozygous *dPNUTS^KG572^* mutant eyes show a weaker phenotype than either *dPNUTS^9B^* or *dPNUTS^13B^*, and this can be enhanced by loss of one copy of *PP187B*.

Since salivary glands from 1^st^ instar *dPNUTS* mutant larvae were too small to analyse in squash preparations, we were unable to test whether loss of *dPNUTS* function displaces PP1 from chromosomes. Therefore, to examine whether PP1 is dependent on dPNUTS for its localisation or *vice versa*, we utilised our transgenic overexpression construct *dPNUTS^W726A^*, which exhibits reduced binding to PP1. We reasoned that if PP1 is necessary for dPNUTS localisation we would expect to observe loss of dPNUTS^W726A^ from chromosomes; conversely, if dPNUTS is responsible for recruiting PP1 then overexpressed dPNUTS^W726A^ should stoichiometrically compete with endogenous PNUTS-PP1 complexes for binding to chromosomes resulting in the displacement of PP1. Chromosomal PP1 staining, but not total PP1 levels, was reduced in glands overexpressing *dPNUTS^W726A^* compared to those expressing *dPNUTS^WT^* (Figure 6A). To quantify the effect on PP1 localisation, we performed line scans to measure fluorescence intensity at a readily identifiable site on the X chromosome, where endogenous PP1 and dPNUTS co-localise (Figure 5D). Intensity of PP1 staining at this site on chromosomes from animals overexpressing *dPNUTS^W726A^* was on average reduced 0.6 fold (Figure 6B). Taken together, these data suggest that *dPNUTS* is responsible for targeting PP1 to many distinct chromosomal loci. Anti-Myc staining of ectopically expressed Myc-tagged dPNUTS was of relatively poor quality but, in general there was a comparable distribution of *dPNUTS^W726A^* and *dPNUTS^WT^* in squash preparations (Figure 6C). Levels of *dPNUTS^W726A^* sometimes appeared weaker than *dPNUTS^WT^* but this is accounted for by differences in the quality of squash preparations and a lower expression level of *dPNUTS^W726A^* relative to *dPNUTS^WT^*, as revealed by immunoblotting (Figure 6D). Taken together these data suggest that PP1 binding is not necessary for dPNUTS localization to polytene chromosomes.

### PP1-binding is important for dPNUTS function in tissue development

To elucidate the functional significance of the interaction between PP1 and dPNUTS *in vivo*, we examined whether *dPNUTS^W726A^* was capable of rescuing the reduced eye phenotype exhibited by our *dPNUTS* mutants. *dPNUTS^WT^* rescued the effect of both *dPNUTS^9B^* and *dPNUTS^13B^*. However, ectopic overexpression of *dPNUTS^W726A^* failed to rescue either mutant (Figure 6E), indicating that binding to PP1 is critical for *dPNUTS* function in tissue development. We also took another approach to examine the effect of reducing PP1 activity in *dPNUTS* mutant eyes. For this, we generated flies that were homozygous for *dPNUTS^KG572^*, which resulted in a modest reduction in eye size, and also heterozygous for mutations in *PP187B* that reduce the total PP1 activity by approximately 40% [44]. Reduced eye phenotypes caused by *dPNUTS^KG572^* mutants were dominantly enhanced by *PP187B*, consistent with *dPNUTS* acting as a positive regulator of PP1 function during imaginal disc development (Figure 6F).

### dPNUTS is complexed with and regulates the phosphorylation state of RNA Polymerase II

**Figure 7:**
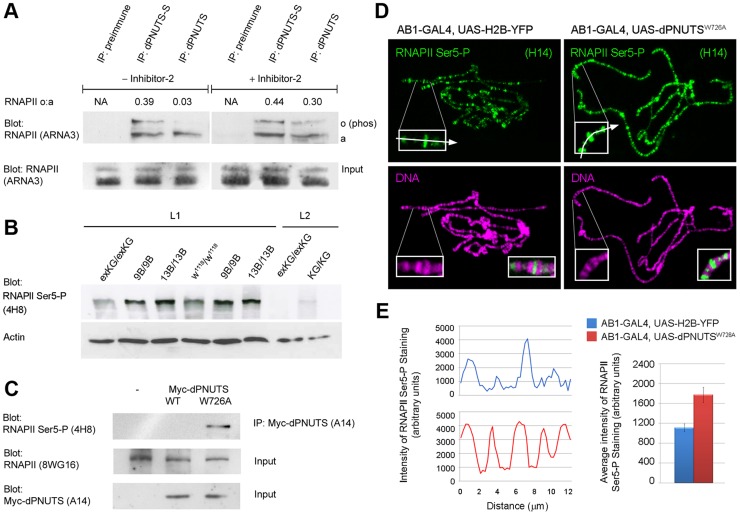
dPNUTS complexes with and regulates RNAPII phosphorylation. A) dPNUTS complexes contain RNAPII and PP1; inhibition of PP1 activity in dPNUTS complexes leads to hyperphosphorylation of RNAPII. dPNUTS-S and dPNUTS were immunoprecipitated (IP) from embryonic nuclear extracts and precipitates were probed with ARNA-3 anti-RNAPII antibody. Lane 1, neither hypo- or hyper-phosphorylated RNAPII (RNAPIIa and RNAPIIo respectively) precipitate with pre-immune serum; Lane 2, both RNAPIIa and RNAPIIo precipitate with dPNUTS-S; Lane 3, RNAPIIa, but almost no RNAPIIo, is detected in dPNUTS precipitates. Lane 4, pre-immune serum does not precipitate RNAPII; Lane 5, Inhibitor 2 does not affect the ability of RNAPIIa and RNAPIIo to associate with dPNUTS-S (compare Lane 2); Lane 6, inhibition of PP1 results in conversion of RNAPIIa to RNAPIIo in dPNUTS precipitates (compare Lane 3). Ratios of RNAPIIa and RNAPIIo levels, as derived from densitometry measurements of the respective bands, are shown above the blots. B) Western Blot showing levels of RNAPII CTD Ser5-P (4H8) in extracts from either 1^st^ (L1) or 2^nd^ (L2) instar larvae of the indicated genotypes: homozygous revertant *dPNUTS^exKG^/dPNUTS^exKG^* (exKG/exKG); homozygous null mutant *dPNUTS^9B^/dPNUTS^9B^* (9B/9B) or *dPNUTS^13B^/dPNUTS^13B^* (13B/13B); isogenic control strain *w^1118^/w^1118^*; homozygous hypomorphic mutant *dPNUTS^KG572^/dPNUTS^KG572^* (KG/KG). 1^st^ instar larval samples from *dPNUTS^9B/9B^* and *dPNUTS^13B/13B^* were independent extracts run in parallel on the same gel. Blot with anti-Actin antibody shows relative loading. C) Precipitation of RNAPII Ser5-P with dPNUTS^W726A^ but not dPNUTS^WT^. dPNUTS complexes from *Drosophila* embryonic nuclear extracts expressing Myc-tagged dPNUTS^WT^ or dPNUTS^W726A^ under the control of *da-GAL4* were isolated by immunoprecipitation with anti-Myc antibody. Control precipitations were performed on *w^1118^* extracts (−). This was followed by immunoblotting with anti-RNAPII CTD Ser5-P (4H8) antibody to test for co-immunoprecipitation. Lower panels show immunoblot analyses of total lysates, confirming the levels of total RNAPII and Myc-dPNUTS. D) Levels of RNAPII CTD Ser5-P (H14) on polytene chromosome squashes from salivary glands expressing either histone-H2B YFP or Myc-*dPNUTS^W726A^* prepared on the same slide to ensure identical staining conditions (H14 staining in green; DNA staining in magenta). Insets are enlarged views of the distal end of the X chromosome. Arrows indicate approximate lines along which quantitation of fluorescence (in E) was performed. E) Representative line scans through the regions illustrated in D, showing levels of RNAPII CTD Ser5-P staining in the two genotypes. Bar graphs represent the average fluorescence in this region from 6 independent images/genotype. Genotypes are indicated by the colour key.

RNAPII has recently been reported to co-precipitate PNUTS from mammalian cell extracts [37]. Given the widespread effects of *dPNUTS* mutations on transcription and its colocalisation with active RNAPII at many transcriptionally active sites on chromosomes, we wondered whether dPNUTS also physically associates with RNAPII complexes. To test this, we immunoprecipitated endogenous dPNUTS from wild type embryo extracts and examined precipitates for the presence of RNAPII. Two RNAPII species, representing unphosphorylated (RNAPIIa) and phosphorylated RNAPII (RNAPIIo), can be detected using an antibody (ARNA-3) that recognises a peptide mapping to central region of RNAPII. Both these forms precipitated with dPNUTS-S, but only RNAPIIA co-precipitated efficiently with dPNUTS (Figure 7A). Since PP1 was previously shown to be capable of dephosphorylating RNAPIIo *in vitro* [14], we wondered whether the pattern of binding we observed was because dPNUTS is capable of binding PP1 and dPNUTS-S is not. To test the role of PP1 in endogenous dPNUTS complexes, we repeated our immunoprecipitations in the presence of Inhibitor-2 (I-2), a specific inhibitor of PP1 [45]. There was no apparent difference in the abundance of RNAPIIa or RNAPIIo in dPNUTS-S precipitates. However, when we precipitated dPNUTS in the presence of I-2, we found reduced levels of RNAPIIa and elevated levels of RNAPIIo (Figure 7A). I-2 selectively targets PP1 over PP2A, which is the next most closely related member of the PPP family of phosphatases [46]. Therefore, we conclude that PP1 is likely to be the major RNAPII phosphatase in these complexes.

Mammalian PNUTS has been reported to bind to Wdr82, which targets RNAPII phosphorylated on Ser5 of its CTD repeats (RNAPII CTD Ser5-P). Although the degree of functional conservation between mammalian and *Drosophila* Wdr82 (dWdr82) has not yet been fully determined, we found that dWdr82 co-precipitated with dPNUTS from *Drosophila* cell extracts indicating that the ability of Wdr82 to bind PNUTS is shared between fly and human orthologues (Figure S6A). This prompted us to assess the effect of *dPNUTS* loss of function on the levels of RNAPII CTD Ser5-P. Using an antibody (4H8) that recognizes the Ser5-phosphorylated C-terminal domain [47], we observed elevated levels of RNAPII CTD Ser5-P in total extracts from *dPNUTS* mutant larval extracts by Western Blotting compared to wild type (*w^1118^*) or revertant (*dPNUTS^exKG^*/*dPNUTS^exKG^*) controls (Figure 7B). Using a panel of independent anti-phospho CTD antibodies [48], [49] we further confirmed the effect of *dPNUTS* loss of function on RNAPII CTD Ser5-P levels. We also observed a modest increase in levels of RNAPII CTD Ser2-P but little or no change in levels of RNAPII CTD Thr4-P or Ser7-P, in mutant extracts (Figure S6B). To test whether *dPNUTS* regulates RNAPII phosphorylation on chromosomes, we generated mutant clones in the salivary gland and examined RNAPII phosphorylation on polytene chromosomes in whole mount preparations. Levels of RNAPII CTD Ser5-P, as detected with an antibody (H14), which recognizes RNAPII Ser5-P in the context of Ser2 phosphorylation [48], were also elevated in this context (data not shown). Interestingly, on wild type polytene chromosome squashes, we observed relatively little co-localisation between dPNUTS and RNAPII Ser5-P (H14) (Figure S7), suggesting that the presence of dPNUTS at chromosomal loci is associated with a reduction of Ser5 phosphorylation at these sites. To confirm the role of dPNUTS-bound PP1, we expressed Myc-tagged *dPNUTS^WT^* and *dPNUTS^W726A^* in embryos, and tested their ability to bind to RNAPII CTD Ser5-P. Immunoprecipitation with anti-Myc antibodies, followed by immunoblotting with anti-RNAPII CTD Ser5-P (4H8) antibody, revealed that RNAPII CTD Ser5-P was only recovered in Myc-*dPNUTS^W726A^* and not Myc-*dPNUTS^WT^* precipitates (Figure 7C), further indicating that dPNUTS-bound PP1 dephosphorylates RNAPII Ser5-P.

### Disruption of PP1-binding results in elevated RNAPII CTD Ser5-P at chromosomal loci

To further test the role of PP1-bound dPNUTS, we examined the effect of ectopic *dPNUTS^W726A^* on RNAPII phosphorylation on polytene chromosome spreads. Since *dPNUTS^W726A^* shows reduced binding to PP1, we predicted that ectopic expression of this mutant form would compete with endogenous PNUTS-PP1 complexes and thereby reduce RNAPII dephosphorylation by PP1. Correspondingly, we found that levels of RNAPII CTD Ser5-P appeared modestly elevated on chromosomes from glands overexpressing *dPNUTS^W726A^* (Figure 7D). To quantitate this effect, we compared the levels of RNAPII CTD Ser5-P staining on chromosomes from larvae with or without ectopic *dPNUTS^W726A^*. Since RNAPII CTD Ser5-P staining was variable from slide to slide, chromosomes from animals over-expressing *dPNUTS^W726A^* were prepared alongside control samples labelled with histone-H2B YFP and stained on the same slides to ensure identical staining conditions between the two samples. Line scans and measurements of average signal intensity at a site at which *dPNUTS^W726A^* displaces endogenous PP1 (Figure 6A,B), indicated an average increase of 1.59 fold in RNAPII Ser5-P on chromosomes from larvae ectopically expressing *dPNUTS^W726A^* compared to wild type animals (Figure 7E). Ectopic *dPNUTS^WT^* on average had no effect on RNAPII Ser5-P staining relative to histone-H2B YFP labeled chromosomes (data not shown). Together, these results indicate that the dPNUTS-PP1 holoenzyme associates with RNAPII and regulates the dephosphorylation of its C-Terminal Domain.

### Misregulation of gene expression, but not RNAPII distribution, is a consequence of disrupting dPNUTS-PP1 binding

Relatively little is known about the effect of RNAPII Ser5 hyperphosphorylation on gene expression, but it has been associated with decreased elongation rate or pausing of RNAPII when it occurs on the body of genes [50], [51]. To assess whether effects on RNAPII occupancy might result from disrupting dPNUTS binding to PP1, we examined the effect of ectopic *dPNUTS^W726A^* on gene expression and the distribution of RNAPII at specific gene loci. Overexpression of *dPNUTS^W726A^* in 3^rd^ instar larvae using *da-GAL4* (*da>dPNUTS^W726A^*) had a similar, but weaker, effect on gene expression to that of *dPNUTS* loss-of-function in 1^st^ instar larvae (Figure S8A). This might be because the transgenic line of *dPNUTS^W726A^* that we used had only a weak dominant-negative effect (animals expressing this construct were viable with *da-GAL4*) and/or because the regulation of some target loci is different at this later developmental stage. Amongst the genes we examined, *ImpL3*, *nop56* and *ACC* were underexpressed, whereas the stress response gene *Thor* was overexpressed in response to ectopic *dPNUTS^W726A^*. Next, we examined the distribution of RNAPII at selected loci by Chromatin Immunoprecipitation (ChIP). For these experiments, chromatin was extracted from *da>dPNUTS^W726A^* or control larvae and precipitated with either mouse IgG or anti-total RNAPII antibody (8WG16). We determined the abundance of precipitated chromatin by qPCR with gene specific primers. Control precipitations with mouse IgG showed a low level of non-specific background in all of these experiments (Figure S8B–E). When we analysed the distribution of RNAPII at selected loci, we did not observe a significant change in the occupancy of total RNAPII at the 5′ ends or coding regions of genes in *da>dPNUTS^W726A^* samples. Together, these results provide evidence of the link between the disruption of PP1 binding to dPNUTS and the misregulation of RNAPII-mediated gene expression, but suggest that changes in gene expression that we have observed may be linked to effects on co-transcriptional processes, such as mRNA capping, rather than transcription *per se*. Indeed, Ser5-P has been shown to bind and stimulate the activity of mammalian capping enzyme (Mce1) [52], [53]. Furthermore, in yeast, lethality resulting from substitution of all CTD Ser5 residues with Ala can be rescued by the tethering of Mce1 to the CTD, suggesting that the essential function of CTD Ser5 is in capping enzyme recruitment [54].

## Discussion

### *dPNUTS* is required for gene expression and for developmental growth

Here we report the functional analysis of *Drosophila* PNUTS, a regulatory subunit of PP1 that is highly conserved between flies and humans. We find that *dPNUTS* is essential for organismal growth, with mutant animals arresting early in larval development. Survival of the null zygotic mutants until the early larval stage is most likely due to perdurance of maternal *dPNUTS* gene products, raising the possibility of additional roles for *dPNUTS* during embryological development that we have not uncovered here. Clonal analysis indicates that *dPNUTS* has a cell autonomous effect on growth, with mutant clones failing to survive unless given a growth advantage. Transcriptomics characterisation of *dPNUTS* mutant animals indicates that the larval arrest phenotype is associated with the underexpression of many RNAPII-dependent genes, including those that normally support developmental growth. Of particular interest in this regard is the significant enrichment of genes involved in cellular metabolism. The underexpression of these genes suggests that an important role of *dPNUTS* during larval growth might be to ensure transcription of highly expressed metabolic pathways responsible for fuelling energy production and generating the macromolecular precursors for RNA and protein synthesis. Metabolic state is monitored in developing epithelia, ensuring that the fittest cells are selected as organ precursors [55]. The failure to compete with wild type neighbours is consistent with an altered metabolic state that is recognised by cell competition, triggering cells to be outcompeted by their neighbours and lost by caspase-dependent apoptosis.

Is the effect on RNAPII-dependent transcription the cause of growth defects? It is conceivable that roles that have been assigned to hPNUTS, e.g. in the DNA damage response and chromatin condensation, are conserved in dPNUTS and these might contribute to the larval lethality exhibited by *dPNUTS* mutants. Indeed the non-identical distribution of dPNUTS and RNAPII on chromosomes suggests that dPNUTS is present in chromatin-associated complexes lacking RNAPII. Notably we do not see any detectable condensation defects in *dPNUTS* mutant clones but we cannot exclude the possibility that *dPNUTS* may also contribute to other processes that underlie tissue growth, such as transcription-independent cell cycle control, as has been reported for other enzymes that regulate CTD phosphorylation, such as FCP1 [56]. Nevertheless, loss of expression of any one of the cell metabolism pathways affected by *dPNUTS* (Table S4) is sufficient to cause larval growth arrest and is likely to explain the failure of *dPNUTS* larvae to grow in size prior to their eventual demise.

### dPNUTS associates with RNAPII at active sites of transcription

Like its mammalian counterpart, we have shown that dPNUTS is a nuclear protein that localises to chromatin during interphase. By utilising larval polytene chromosomes, which are readily visible by light microscopy, we have been able to extend this analysis by determining the distribution of dPNUTS on interphase chromosomes *in situ*. These analyses show co-localisation of dPNUTS with many transcriptionally active sites marked with RNAPII, suggesting that the widespread changes in gene expression that we observe upon loss of *dPNUTS* function are likely to be due to the direct involvement of *dPNUTS* in RNAPII-mediated transcriptional regulation. Correspondingly, we find that dPNUTS is complexed to the large subunit of RNAPII in cell extracts. However, it is important to note that not all RNAPII sites stain for dPNUTS (and *vice versa*) and the relative amounts of the two proteins vary widely amongst these sites. This suggests that the association of dPNUTS with RNAPII, or with associated factors, which may affect the availability of the dPNUTS epitope for detection by our antibody, may be differentially regulated. PNUTS contains a number of conserved macromolecular-interaction domains, which have led to the suggestion it might serve as a multivalent adapter protein. However, it has not yet been established to what extent the known interactors, Tox4 and Wdr82 aid in the recruitment of PNUTS to chromosomal loci. These issues will require investigation of the genome-wide sites of dPNUTS binding, as well as identification and comprehensive characterisation of dPNUTS-interacting proteins and their role in dPNUTS recruitment.

### dPNUTS-PP1 regulates the phosphorylation state of RNAPII

Since we found that PP1-binding is necessary for dPNUTS function, we reasoned that dPNUTS affects transcription by targeting PP1 to specific substrates on chromosomes. Several lines of evidence indicate that one important target of dPNUTS-PP1 in this context is the CTD of RNAPII: i) dPNUTS is complexed with RNAPII in nuclear extracts and regulates RNAPII CTD phosphorylation in a PP1-dependent manner; ii) RNAPII CTD Ser5-P levels are elevated in *dPNUTS* mutant larval extracts and tissues; iii) dPNUTS colocalises with PP1 and RNAPII on chromosomes; iv) ectopic expression of a mutant version of dPNUTS that displaces PP1 from polytene chromosomes results in elevated RNAPII CTD Ser5-P levels on chromosomes. dPNUTS-PP1 appears to preferentially target Ser5-P of the CTD as we observed only a modest effect on Ser2-P levels and no effect on phosphorylation of other RNAPII-CTD residues in *dPNUTS* mutant larval extracts by Western blotting (Figure S6B). However, PNUTS/PP1 is not the only PP1 holoenzyme that has been implicated in regulation of RNAPII phosphorylation [37], raising the possibility that different PP1 holoenzymes possess different RNAPII CTD specificities.

Changes in the pattern of gene expression that we have observed in *dPNUTS* mutant animals are correlated with the normal expression level of the affected transcripts; these changes may also reflect the spatial distribution of *dPNUTS* expression during development. During embryogenesis we observed that the levels of *dPNUTS* expression in the gut and the ventral nerve cord correlates with stages in which these tissues are undergoing periods of rapid expansion and development. In an analogous fashion to SCP1, which restricts RNAPII dephosphorylation of neuronal genes to non-neuronal cells by virtue of its expression pattern [11], the enrichment of *dPNUTS* in proliferating tissues may function to promote expression of highly expressed transcripts, such as those involved in cellular metabolism, in these tissues, to support their development. In mammals, the gradual decrease from a high level of PNUTS during embryogenesis to a relatively low level in adults has been taken to imply that PNUTS could play a role in cortical development [22], but could equally reflect a requirement during growth of developing tissues. Notably, PNUTS is not found in some metazoans such as *C.elegans*, where strictly controlled cell lineage determines tissue architecture. An evolved function of PNUTS might therefore be to support proliferative states in organisms where compensatory mechanisms such as cell competition are at play.

*How do dPNUTS and RNAPII hyperphosphorylation regulate gene expression?* Studies of other enzymes that control CTD phosphorylation state indicate that maintaining correct levels of CTD phosphorylation is critical for normal levels of transcription and that hyperphosphorylation of RNAPII can increase or reduce gene expression depending on what stage of the transcriptional cycle phosphorylation is affected. For instance, FCP1 targets Ser2-P *in vivo* [57] and is thought to recycle RNAPII after the complex has dissociated from the transcribed region [58]. Correspondingly, conditional knockout of FCP1 in yeast results in a global defect in transcription affecting 77% of genes [59]. SCP1 and Ssu72 both target Ser5-P [16], [60], but have contrasting roles in transcriptional regulation: knockdown of SCP1 unmasks neuronal gene expression, indicating it normally acts as a transcriptional repressor [11], whilst Ssu72 facilitates transcription by promoting the elongation stage of the transcription cycle [61]. ChIP experiments from larvae expressing *dPNUTS^W726A^* suggest that displacement of PP1 binding to dPNUTS does not result in accumulation of RNAPII on the coding region of affected loci. The precise mechanisms of how loss of *dPNUTS* function and RNAPII hyperphosphorylation disrupt gene expression require further investigation. However, we might expect processes dependent on normal CTD phosphorylation, including RNA processing, transcription-coupled chromatin modification and transcription-associated homologous recombination [4], to be affected. In this regard, it is notable that inhibition of TFIIH kinase activity, which phosphorylates promoter-bound RNAPII at Ser5, predominantly affects mRNA capping and stability rather than transcription *per se* [62]–[64].

In summary, the analysis of *dPNUTS* described here reveals an important function for this evolutionarily conserved chromatin-associated protein, via association with PP1, in the regulation of RNAPII phosphorylation and the appropriate expression of genes during larval development, which support organismal growth. These findings provide insight into the role of PNUTS and RNAPII phosphorylation during normal development, and may also be of relevance to the understanding of aberrant gene expression patterns observed in disease processes and ageing.

## Materials and Methods

### Fly strains

*Drosophila melanogaster* stocks were kept at 18°C or 25°C on standard agar-cornmeal-yeast medium. Genotypes are provided in *Text S1*.

### Isolation and characterisation of *dPNUTS* null alleles

Isolation of a null allele of *dPNUTS* by *P* element excision from *dPNUTS^KG^* was carried out by crossing *w*; *dPNUTS^KG^*/*CyO*, *P*(*Delta2-3*) males to *y*, *w*; *Tft*/*CyO* females. From each cross, a single *w* revertant male in which the *P* element was excised, was individually crossed back to *w*; *Tft*/*CyO* females. To determine the molecular lesion in excisions, genomic DNA surrounding the original *dPNUTS^KG^* insertion site was amplified from heterozygous mutants by PCR using flanking primers (see *Text S1*) and sequenced. For genetic complementation tests, a 9.1 kb *Bam*HI restriction fragment from P1 clone DS02684, which contains all of the transcribed *dPNUTS* sequence, was subcloned into the *Bam*HI site in pW8 and injected into flies. Details of the growth arrest experiment can be found in *Text S1*.

### Ectopic expression of wild type *dPNUTS* and *dPNUTS^W726A^*

Full-length cDNAs for dPNUTS-S and PNUTS-L cloned into pNB40 were isolated from a 3^rd^ instar larval library (see *Text S1*). *dPNUTS^W726A^* was generated by PCR-based site-directed mutagenesis. For ectopic expression in flies, full-length d*PNUTS^WT^* and *dPNUTS^W726A^* were subcloned into pUAS-HM, a modified of pUAST that contains an N-terminal 3× His 6× Myc (HM) tag. *UAS-HM-PNUTS* flies were made by *P* element-mediated germline transformation into a *w^1118^* stain by Genetic Services Inc. (Cambridge, MA). Tagged *dPNUTS^WT^* and *dPNUTS^W726A^* were ectopically expressed ubiquitously using *da-GAL4* or in salivary glands using *AB1-GAL4*.

### RNA *in situ* hybridisation

pNB40-dPNUTS clones were used to generate Digoxigenin (DIG)-labelled RNA probes. RNA *in situ* hybridisation was essentially performed as previously described [45], [65]. Following hybridization, DIG-labelled probes were detected with an alkaline phosphatase conjugated anti-digoxygenin antibody in the presence of Nitro-blue tetrazolium salt (NBT) and X-phosphate/5-Bromo-4-chloro-3-indolyl-phosphate (BCIP).

### RNA extraction and qRT-PCR

RNA was extracted using the Qiagen RNeasy Mini kit and was reverse transcribed using the High Capacity cDNA Reverse Transcription kit (Applied Biosystems). Quantitative PCR was performed following the incorporation of SYBRGreen (using the Applied Biosystem StepOnePlus Real Time PCR System). Primers are described in *Text S1*. All samples were normalized to 18S RNA. The ΔΔ*C_T_* method was used for the calculation of the relative abundances [66].

### RNA-seq and bioinformatics

RNA from approximately 5000 1^st^ instar larvae/genotype was extracted using the Qiagen RNeasy Mini kit following the manufacturer's instructions. Total RNA quality and quantity was verified on a NanoDrop1000 spectrophotometer (Thermofisher) and Bioanalyzer 2100. mRNA was polyA selected using Dynabeads mRNA Purification Kit for mRNA Purification from Total RNA Preps (Invitrogen). The libraries were prepared according to the SOLiD Total RNA-Seq Kit protocol (Part Number 4452437 Rev. A, Applied Biosystems). RNA samples were sequenced on an AB SOLiD sequencing platform with v4 chemistry, generating single-end 50 bp colour-space reads. More than 93M reads were generated for each sample. Reads were filtered for quality and mapped onto the dm3 *D. melanogaster* reference genome [67], [68] using TOPHAT [69]. Only uniquely mapped reads were retained for analysis and reported as a BAM [70] file. Cufflinks [71] software took the BAM files to calculate expressions levels for annotated and predicted transcripts using FPKM (fragments per kilobase of transcript per million fragments mapped) values. Differentially expressed genes in the *dPNUTS* mutants were defined as genes with <0.67 or >1.5 fold change relative to controls. A significance threshold of 1 FPKM [72] was also applied. To analyse the enrichment of the genes belonging to specific biological processes, genes differentially expressed in both *dPNUTS* mutants were further analysed by Database for Annotation, Visualization and Integrated Discovery (DAVID) (http://david.abcc.ncifcrf.gov/) against the *D. melanogaster* database. To increase the reproducibility, enrichment of gene function was identified with EASE score ≤0.001, which is a conservative adjustment to Fisher exact probability, and a fold change enrichment (FE)≥1.5 in all samples. The GO terms were hierarchically classified using AMIGO. Human orthologues of differentially expressed genes were identified by BioMart (www.biomart.org) and used to reconstruct functional networks and predict upstream regulators using Ingenuity IPA (Ingenuity Systems Inc.), see *Text S1* for details.

### Immunoprecipitation from *Drosophila* extracts and immunoblotting

Immunoprecipitation from 2–18 hr old *Oregon R Drosophila* embryonic nuclear extracts was performed as in [73], with minor modifications (see *Text S1*), using the following primary antibodies: rabbit anti-Myc (A14, Santa Cruz Biotechnology, 1∶100); mouse anti-Myc (9E10, 1∶50); guinea pig anti-dPNUTS and anti-dPNUTS-S (1∶10). The following primary antibodies were used for Western Blotting: mouse anti-RNAPII (ARNA-3, Research Diagnostics/Millipore, 1∶500), which recognizes both phosphorylated and unphosphorylated forms of RNAPII; mouse anti-RNAPII Ser5-P (4H8, Active Motif, 1∶1000); purified rabbit anti-PP1 (1∶500); rabbit anti-Myc (A14, Santa Cruz Biotechnology, 1∶500); mouse anti-Actin (C4, Millipore, 1∶5000). For quantitation, X-ray film was digitized with an ImageQuant biomolecular imager (GE Healthcare) and quantified using ImageJ (http://rsbweb.nih.gov/ij/).

### Immunostaining of wing discs and whole mount salivary glands

Tissues were fixed and stained using standard approaches (see *Text S1*) with the following primary antibodies: rabbit anti-Cleaved Caspase-3 (Cell Signalling Technology, 1∶100); mouse anti-Discs large (Developmental Studies Hybridoma Bank, 1∶100); rabbit anti-Myc (A14, Santa Cruz Biotechnology, 1∶100); mouse anti-phospho-Histone H3 (Millipore, 1∶500). TO-PRO-3 (Invitrogen, 1∶1000) was used to visualise DNA.

### Immunostaining of polytene chromosomes

Polytene chromosome squashes were prepared as reported previously [74] (see also *Text S1*) and stained with the following primary antibodies: guinea pig anti-dPNUTS (1∶30); rabbit anti-PP1 (1∶50); mouse anti-RNAPII Ser2-P (H5, Covance, 1∶50); mouse anti-RNAPII Ser5-P (H14, Covance, 1∶50); rabbit anti-Myc (A14, Santa Cruz Biotech, 1∶100). For DNA staining, slides were incubated with either DAPI or TO-PRO-3.

### Image analysis and quantitation

Images were captured on Zeiss 510 and 710 Confocal Microscopes equipped with 405 nm, 488 nm, 561 nm and 633 nm lasers using a Plan Apochromat 40x/1.3NA oil immersion objective. Images were imported to Adobe Photoshop and adjusted for brightness and contrast uniformly across entire fields. Projected images of wing discs in XY were generated using ImageJ. XZ projections were generated using the Cut function in Zen 2011 (Zeiss). Line scans of polytene chromosomes were generated using ImageJ. For this analysis, we imaged a region at end of the X chromosome that could be reliably identified on chromosomes from multiple squashes. Images were taken with identical microscope and laser settings, with signal intensities below the level of saturation. The mean intensity of RNAPII Ser5-P and PP1 fluorescence was determined for each genotype by calculating the average fluorescence intensity through the center of unprocessed images of the same chromosomal region from 6 samples, parallel to the long axis of the structure.

### GenBank accession numbers

The accession numbers for the *dPNUTS* and *dPNUTS-S* nucleotide sequences reported in this paper are AJ580979 and AJ580980, respectively.

## Supporting Information

Figure S1 Sequence comparison of dPNUTS and related proteins. A) Schematic representation of domains in human PNUTS (hPNUTS) and dPNUTS: Region similar to Domain 1 of TFIIS (and the corresponding domain in Elongin A); Ser-rich region; Central region, highly conserved in hPNUTS and dPNUTS containing a canonical PP1 binding motif; CCCH zinc-finger typical of NUP/Tis11 proteins. The positions of introns (arrowheads) in the coding regions are indicated. Identical intron-exon boundaries are shown with connecting arrows. B) Table of % identity and similarity of hPNUTS, Elongin A and Tis11 in the different domains relative to dPNUTS. NA, not applicable. Pairwise comparisons were performed using ALIGN [77].
(TIF)

Figure S2 Specificity of the dPNUTS antibody for immunofluorescent staining of polytene chromosomes. Chromosome squashes from salivary glands expressing either *histone H2B-YFP* (in green) or *dPNUTS RNAi* stained on the same slide for dPNUTS (in red) and DNA (in magenta). Levels of dPNUTS were greatly reduced on chromosomes from *dPNUTS RNAi* glands.
(TIF)

Figure S3 A) Expanded images of clones in panels K–P of Figure 3, showing DNA and GFP channels for each image. B) Magnified image of panel L of Figure 3, with cross section through a section of the epithelium containing a large *dPNUTS* mutant clone, which shows normal distribution of nuclei compared to neighbouring heterozygous (GFP positive) cells. In contrast, a rare *M, GFP*/*M, GFP* twinspot is located at the basal face of the epithelium and is being extruded.
(TIF)

Figure S4 Venn diagram showing overlap between differentially expressed up- and down-regulated genes in *dPNUTS* mutants.
(TIF)

Figure S5 Gene ontology (GO) term enrichment of the genes under-expressed (A) and over-expressed (B) in *dPNUTS^9B^*/*dPNUTS^9B^* and *dPNUTS^13B^*/*dPNUTS^13B^* mutant larvae relative to abundance of GO terms for all genes in the genome as determined by DAVID. The top GO categories for each gene set are grouped according to their hierarchical relationships along with the number of genes affected in that category, the total number of genes in that category (in parentheses), and the statistical significance of the match.
(TIF)

Figure S6 A) dPNUTS binds dWdr82 in S2 cell extracts. Cells were transfected with constructs expressing Flag-Myc-dWdr82 or GFP-dPNUTS-Myc or both. Ectopic dPNUTS was precipitated using GFP-Trap beads. Western Blotting with anti-Myc antibodies revealed the presence of ectopic GFP-dPNUTS-Myc in precipitates. Flag-Myc-dWdr82 co-precipitated with GFP-dPNUTS-Myc, but not from cells lacking ectopic dPNUTS. IN = Input (total lysate), NB = Non-bound, and IP = immuno-precipitated. B) Western Blot showing levels of RNAPII CTD Ser2-P, Thr4-P, Ser5-P, or Ser7-P in extracts from homozygous revertant *dPNUTS^exKG^*/*dPNUTS^exKG^* (exKG/exKG) and homozygous null mutant *dPNUTS^9B^*/*dPNUTS^9B^* (9B/9B) 1^st^ instar larvae. mAb identity is indicated in parenthesis. Relative levels in the two conditions, as derived from densitometry measurements of the respective bands, are shown below the blots. C) Published conditions of recognition of phospho-CTD by mAbs, reproduced from [48], [49]. Phosphorylation of red amino acids results in full or partial inhibition of mAb binding, whereas phosphorylation of other Tyr, Ser or Thr residues does not.
(TIF)

Figure S7 Polytene chromosomes from salivary gland squashes stained with dPNUTS and RNAPII Ser5-P (H14) antibodies. Merging of the green signal representing RNAPII Ser5-P with the red signal representing dPNUTS identifies sites where these two proteins co-localize. Insets, boxes 1–4, show enlarged view of chromosome regions. The relative signals of dPNUTS and RNAPII Ser5-P vary between sites, but only a minority of dPNUTS loci colocalize with RNAPII Ser5-P staining (indicated with arrows).
(TIF)

Figure S8 A) Expression levels of the indicated genes in larvae expressing *dPNUTS^W726A^* under the control of *da-GAL4* relative to control larvae, as determined by qRT-PCR. Error bars represent the SE (n≥3 biological replicates). B–E) Chromatin immunoprecipitation (ChIP) analyses of the indicated genes from 3^rd^ instar larval extracts using anti-total RNAPII (8WG16) antibody and mouse IgG antibody. Immunoprecipitated DNA was amplified by qPCR. The distribution at four loci (*Thor*, *ImpL3*, *nop56* and *ACC*) was evaluated using primers positioned at the start (S) and middle (M), of the transcribed sequences. Percent input is the amount of precipitated DNA relative to input DNA. Error bars represent the SE of the mean (n≥3 biological replicates).
(TIF)

Table S1 Rescue of *dPNUTS* mutant lethality by genomic transgene. Expected and observed genotype frequencies of adult progeny from complementation crosses with two independent insertions of a *dPNUTS* wild type trangene (n≥350 progeny/cross).
(DOCX)

Table S2 Gene Ontology (GO) classification determined by DAVID. Biological process categories from GO analysis that are significantly overrepresented among the genes for which the expression was either decreased (downregulated worksheet) or increased (upregulated worksheet) in the *dPNUTS* mutants. Only the categories with a minimum of 4 genes per category and an EASE score ≤0.001 were considered.
(XLSX)

Table S3 Comparison of Gene Ontology (GO) outputs from DAVID and EASE. Shown are GO categories that were enriched amongst genes that are differentially expressed (DE) in *dPNUTS* mutants when compared against all genes in the genome (DAVID) or against genes expressed in matched *w^1118^* controls (EASE). GO categories returned by the two approaches were not always identical because the programs used different versions of the *D. melanogaster* genome annotation for comparison (DAVID was updated Sept 2009; EASE used FlyBase annotation release 5.46 from July 2012).
(XLS)

Table S4 Ingenuity transcription factor analysis. The table shows the IPA predicted ‘upstream regulators’ for up- and down-regulated differentially expressed genes, ranked by an application of a z-score algorithm. Genes from each predicted regulator ‘pool’ present in the analyzed dataset are listed in a ‘Target molecules’ column.
(XLSX)

Table S5 Comparison of RNA-Seq and qRT-PCR data, showing log_2_ fold change in expression of the indicated loci in *dPNUTS* mutants relative to control.
(DOCX)

Text S1 Additional information including detailed genotypes and primer sequences as well as methodology for supplementary figures.
(DOCX)
