# Supplementary figures and images for: PNUTS/PP1 Regulates RNAPII-Mediated Gene Expression and Is Necessary for Developmental Growth
Source: PLoS Genet. 2013 Oct 31;9(10):e1003885. doi: 10.1371/journal.pgen.1003885 (PMC3814315; doi:10.1371/journal.pgen.1003885)

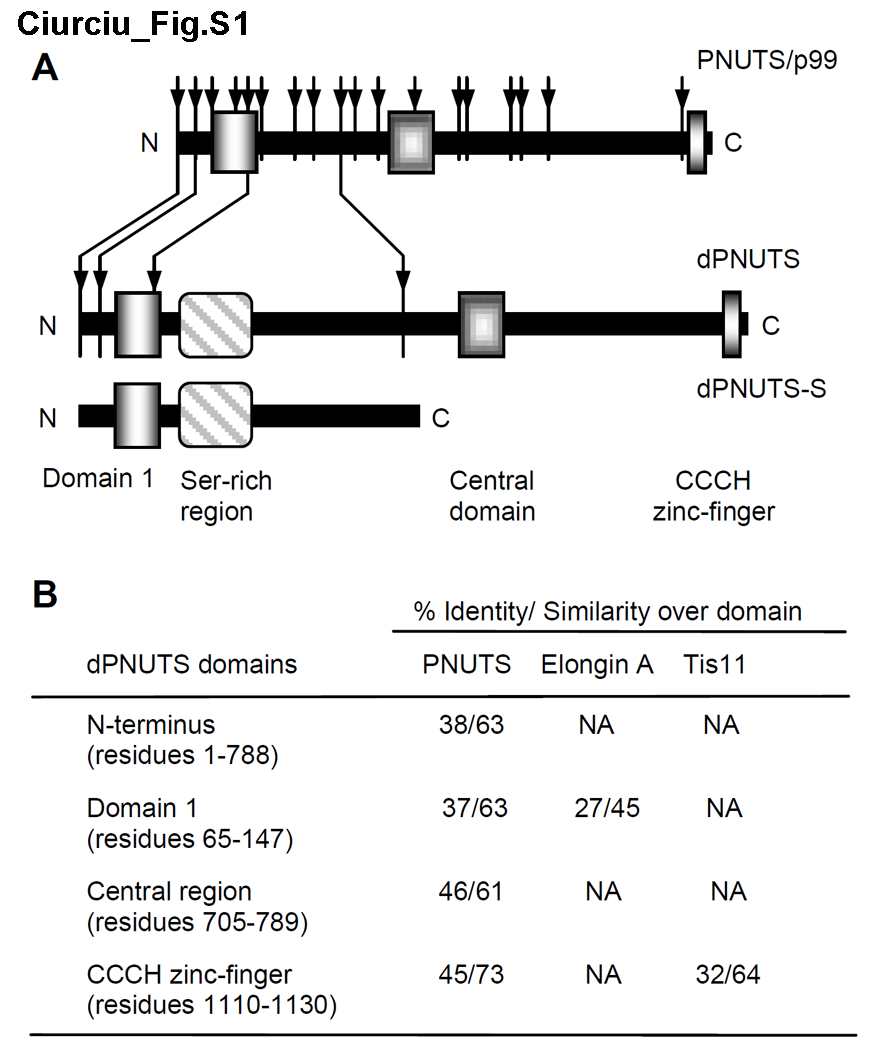

Supplement: Figure S1 — Sequence comparison of dPNUTS and related proteins. A) Schematic representation of domains in human PNUTS (hPNUTS) and dPNUTS: Region similar to Domain 1 of TFIIS (and the corresponding domain in Elongin A); Ser-rich region; Central region, highly conserved in hPNUTS and dPNUTS containing a canonical PP1 binding motif; CCCH zinc-finger typical of NUP/Tis11 proteins. The positions of introns (arrowheads) in the coding regions are indicated. Identical intron-exon boundaries are shown with connecting arrows. B) Table of % identity and similarity of hPNUTS, Elongin A and Tis11 in the different domains relative to dPNUTS. NA, not applicable. Pairwise comparisons were performed using ALIGN [77]. (TIF) [file pgen.1003885.s001.tif]

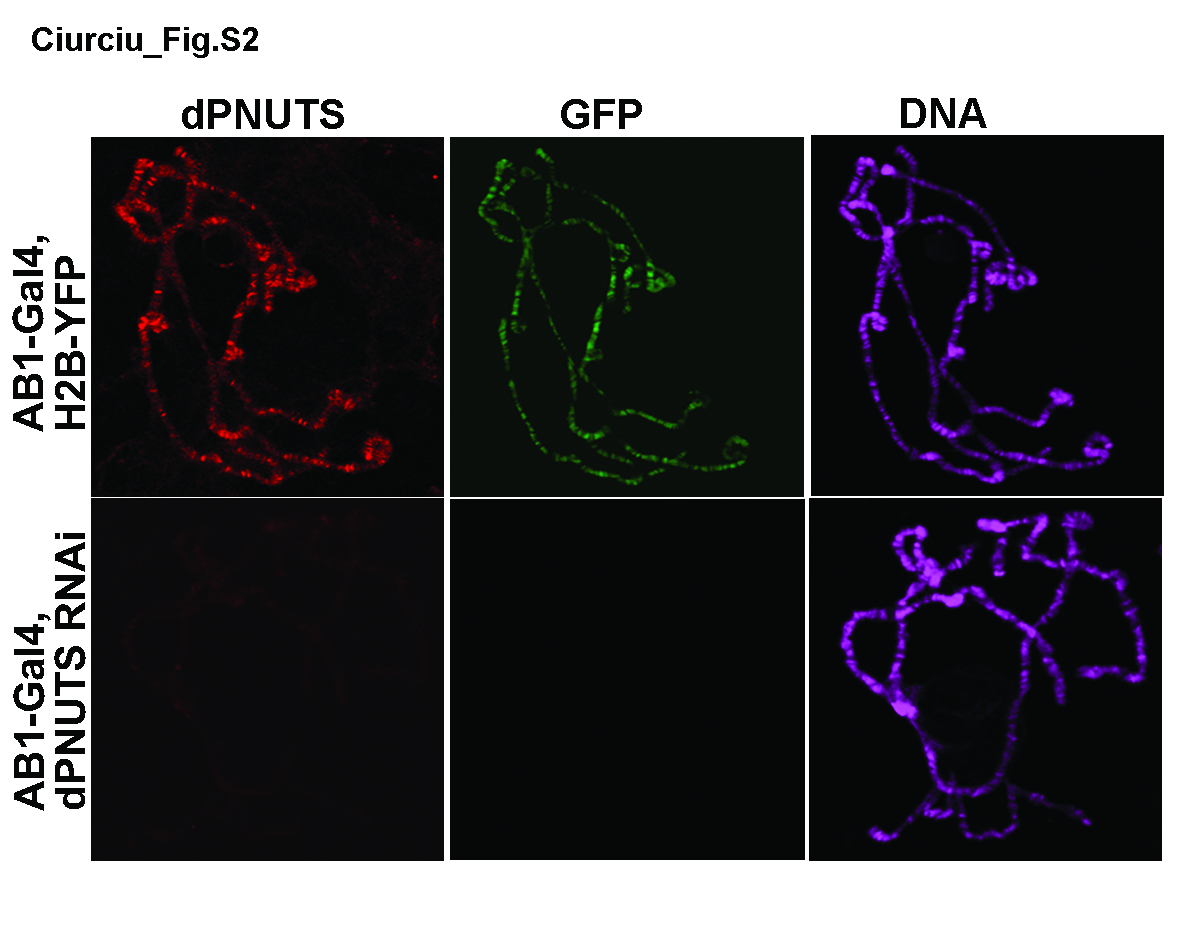

Supplement: Figure S2 — Specificity of the dPNUTS antibody for immunofluorescent staining of polytene chromosomes. Chromosome squashes from salivary glands expressing either histone H2B-YFP (in green) or dPNUTS RNAi stained on the same slide for dPNUTS (in red) and DNA (in magenta). Levels of dPNUTS were greatly reduced on chromosomes from dPNUTS RNAi glands. (TIF) [file pgen.1003885.s002.tif]

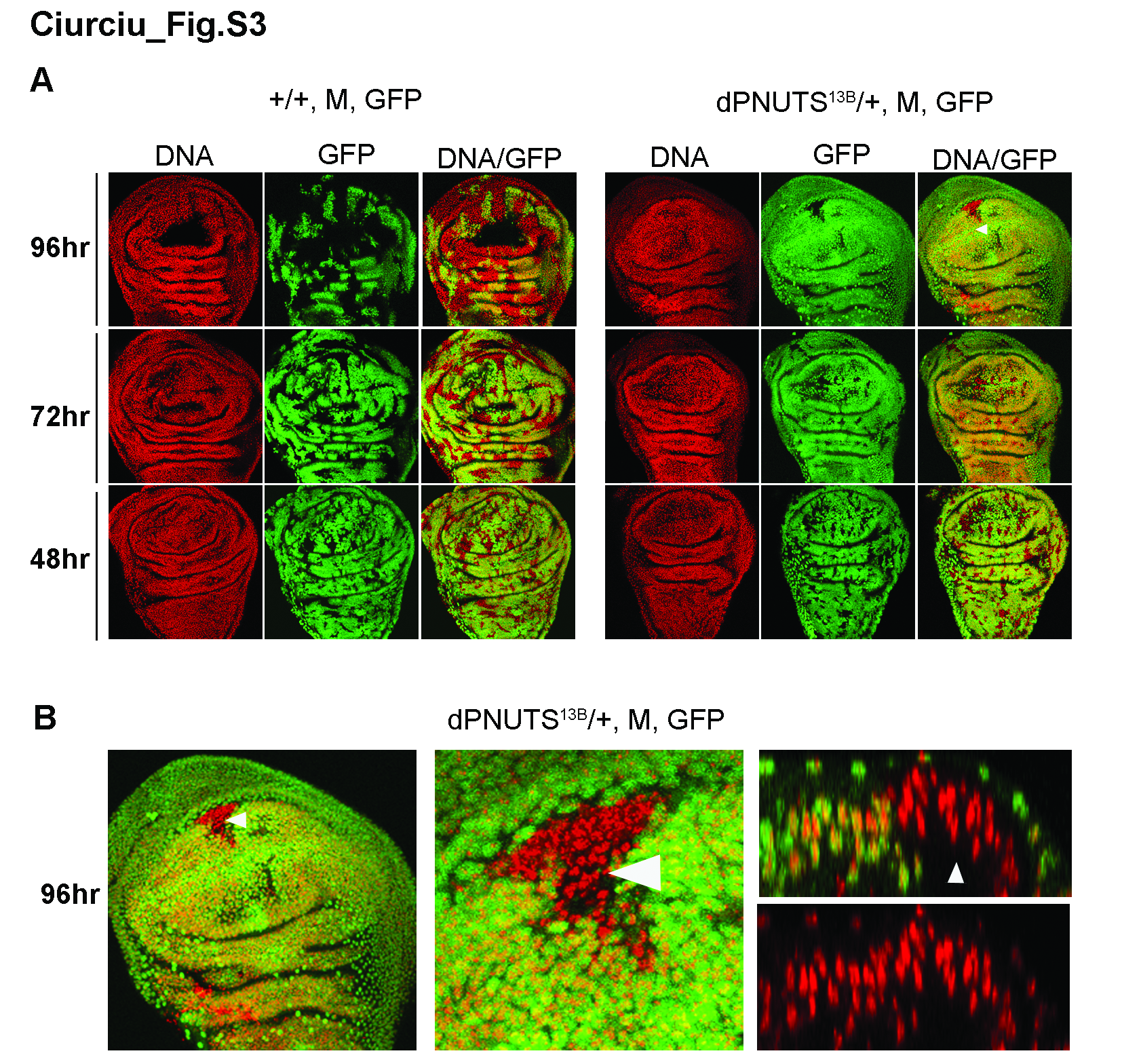

Supplement: Figure S3 — A) Expanded images of clones in panels K–P of Figure 3, showing DNA and GFP channels for each image. B) Magnified image of panel L of Figure 3, with cross section through a section of the epithelium containing a large dPNUTS mutant clone, which shows normal distribution of nuclei compared to neighbouring heterozygous (GFP positive) cells. In contrast, a rare M, GFP/M, GFP twinspot is located at the basal face of the epithelium and is being extruded. (TIF) [file pgen.1003885.s003.tif]

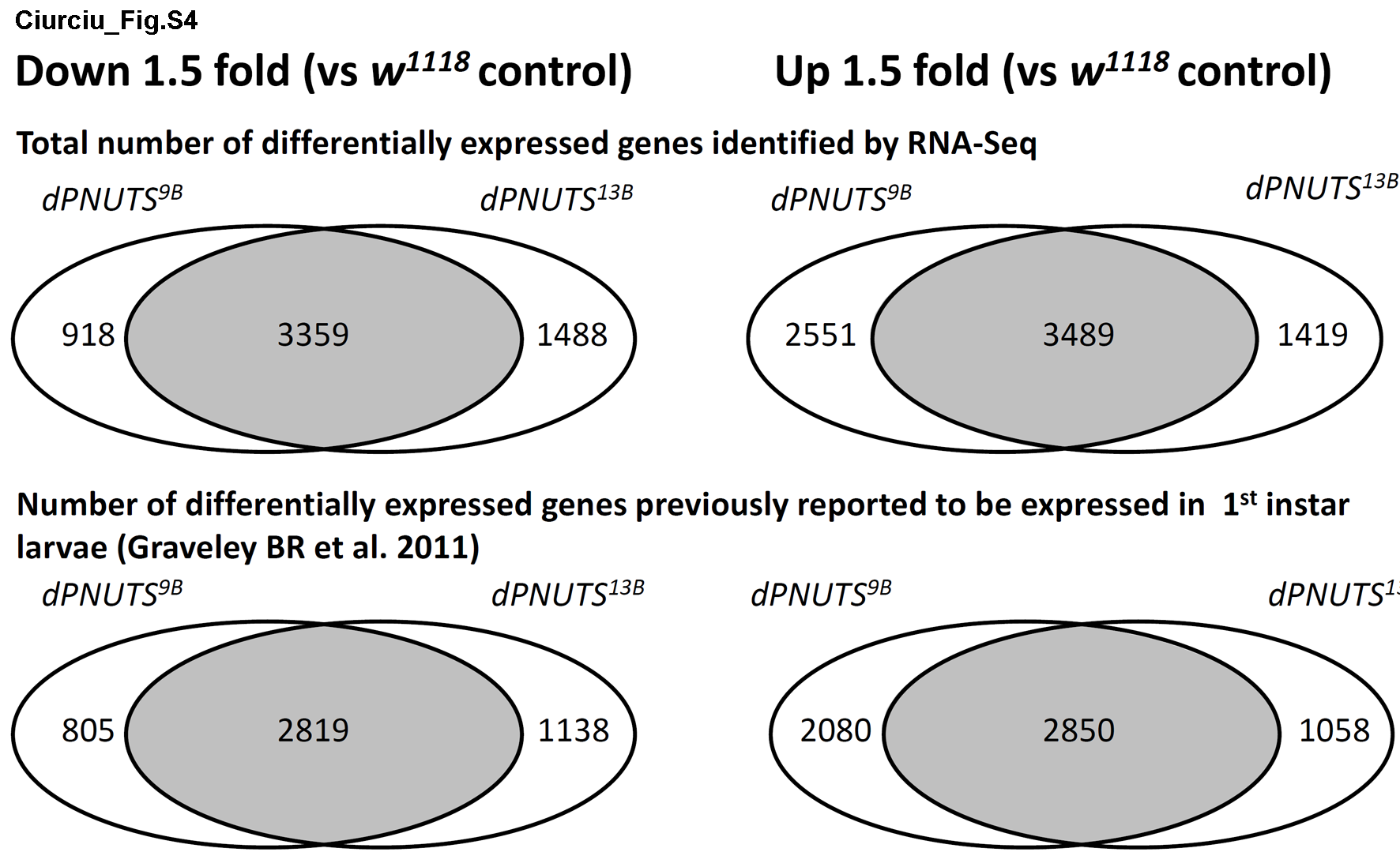

Supplement: Figure S4 — Venn diagram showing overlap between differentially expressed up- and down-regulated genes in dPNUTS mutants. (TIF) [file pgen.1003885.s004.tif]

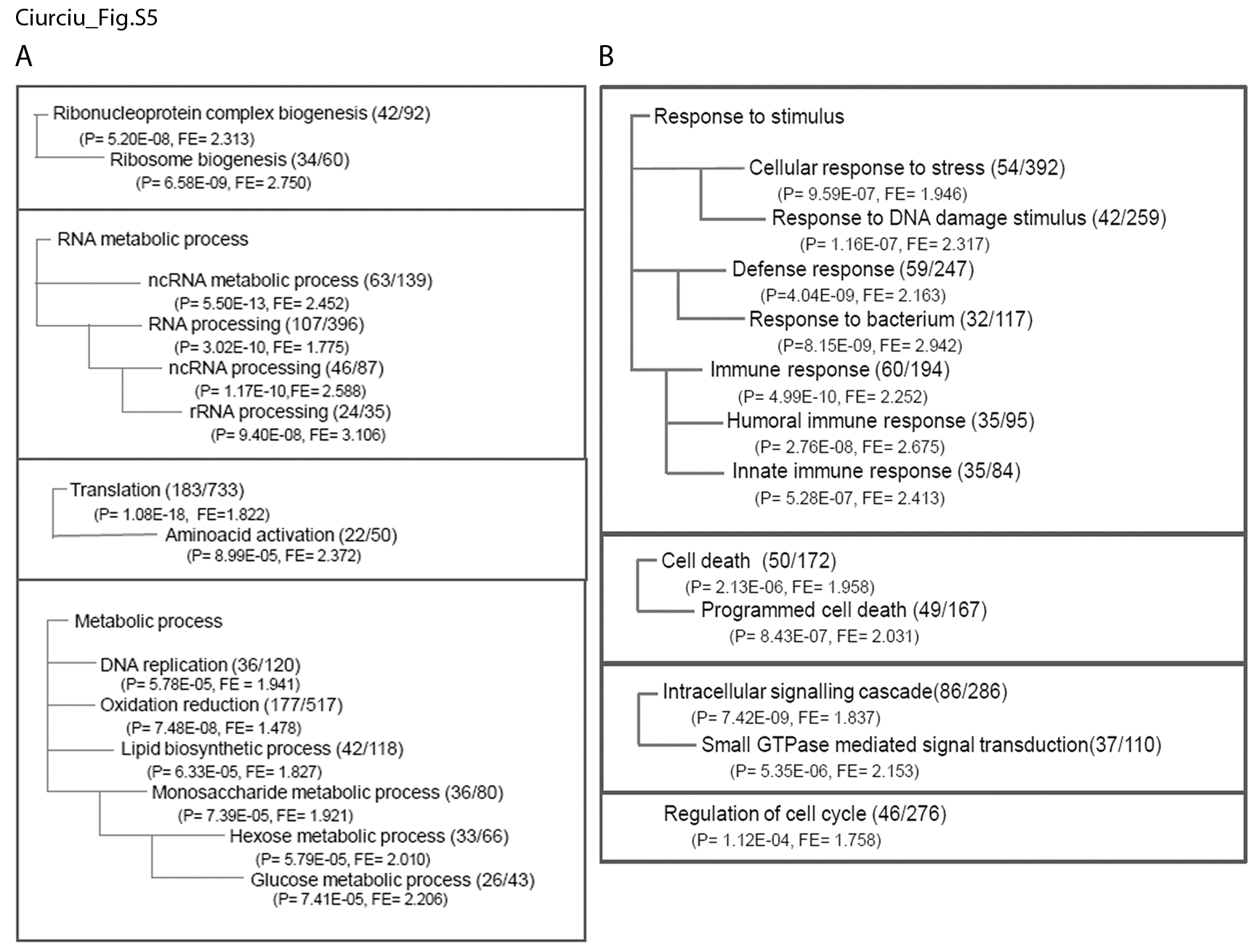

Supplement: Figure S5 — Gene ontology (GO) term enrichment of the genes under-expressed (A) and over-expressed (B) in dPNUTS9B/dPNUTS9B and dPNUTS13B/dPNUTS13B mutant larvae relative to abundance of GO terms for all genes in the genome as determined by DAVID. The top GO categories for each gene set are grouped according to their hierarchical relationships along with the number of genes affected in that category, the total number of genes in that category (in parentheses), and the statistical significance of the match. (TIF) [file pgen.1003885.s005.tif]

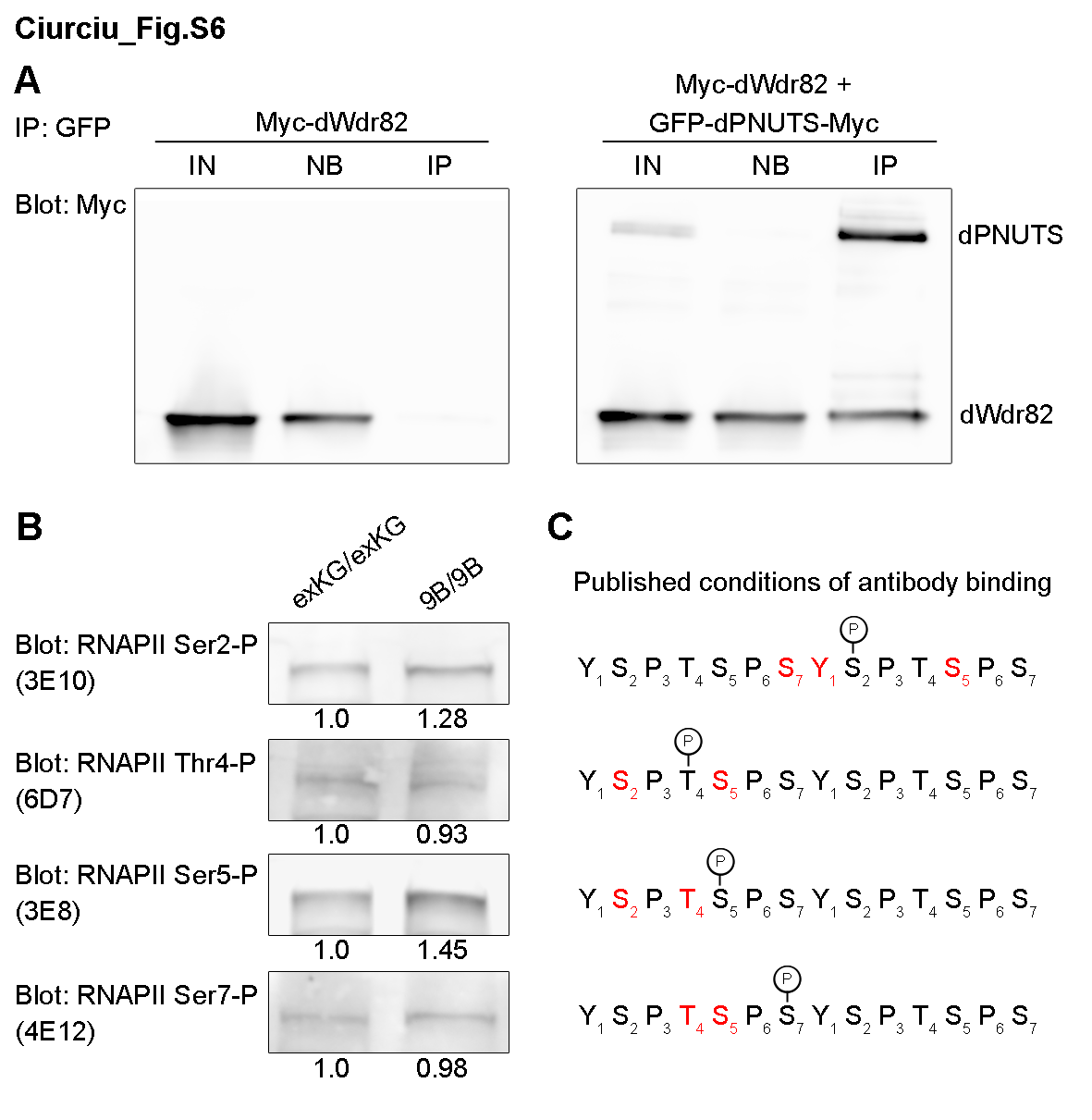

Supplement: Figure S6 — A) dPNUTS binds dWdr82 in S2 cell extracts. Cells were transfected with constructs expressing Flag-Myc-dWdr82 or GFP-dPNUTS-Myc or both. Ectopic dPNUTS was precipitated using GFP-Trap beads. Western Blotting with anti-Myc antibodies revealed the presence of ectopic GFP-dPNUTS-Myc in precipitates. Flag-Myc-dWdr82 co-precipitated with GFP-dPNUTS-Myc, but not from cells lacking ectopic dPNUTS. IN = Input (total lysate), NB = Non-bound, and IP = immuno-precipitated. B) Western Blot showing levels of RNAPII CTD Ser2-P, Thr4-P, Ser5-P, or Ser7-P in extracts from homozygous revertant dPNUTSexKG/dPNUTSexKG (exKG/exKG) and homozygous null mutant dPNUTS9B/dPNUTS9B (9B/9B) 1st instar larvae. mAb identity is indicated in parenthesis. Relative levels in the two conditions, as derived from densitometry measurements of the respective bands, are shown below the blots. C) Published conditions of recognition of phospho-CTD by mAbs, reproduced from [48], [49]. Phosphorylation of red amino acids results in full or partial inhibition of mAb binding, whereas phosphorylation of other Tyr, Ser or Thr residues does not. (TIF) [file pgen.1003885.s006.tif]

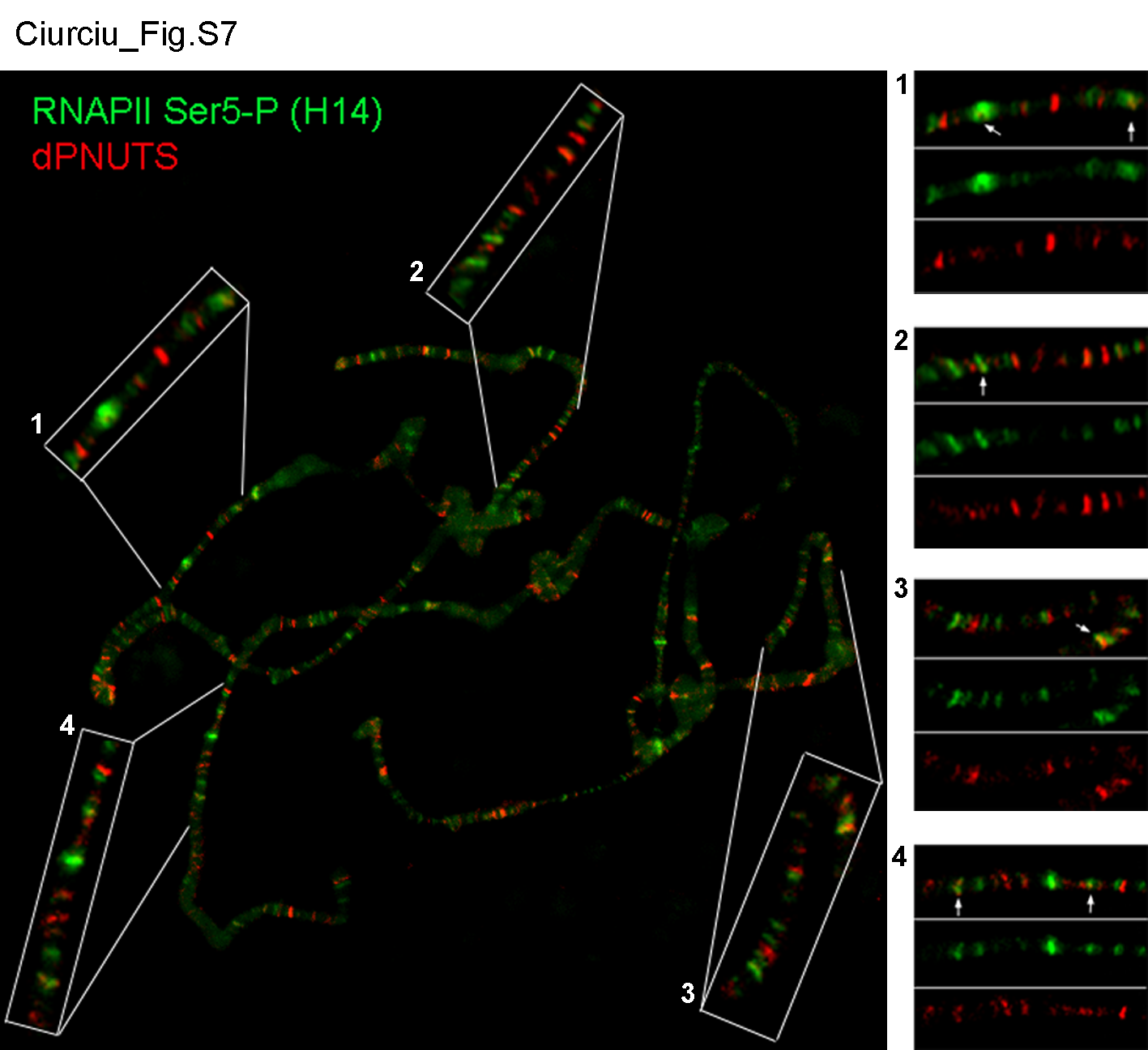

Supplement: Figure S7 — Polytene chromosomes from salivary gland squashes stained with dPNUTS and RNAPII Ser5-P (H14) antibodies. Merging of the green signal representing RNAPII Ser5-P with the red signal representing dPNUTS identifies sites where these two proteins co-localize. Insets, boxes 1–4, show enlarged view of chromosome regions. The relative signals of dPNUTS and RNAPII Ser5-P vary between sites, but only a minority of dPNUTS loci colocalize with RNAPII Ser5-P staining (indicated with arrows). (TIF) [file pgen.1003885.s007.tif]

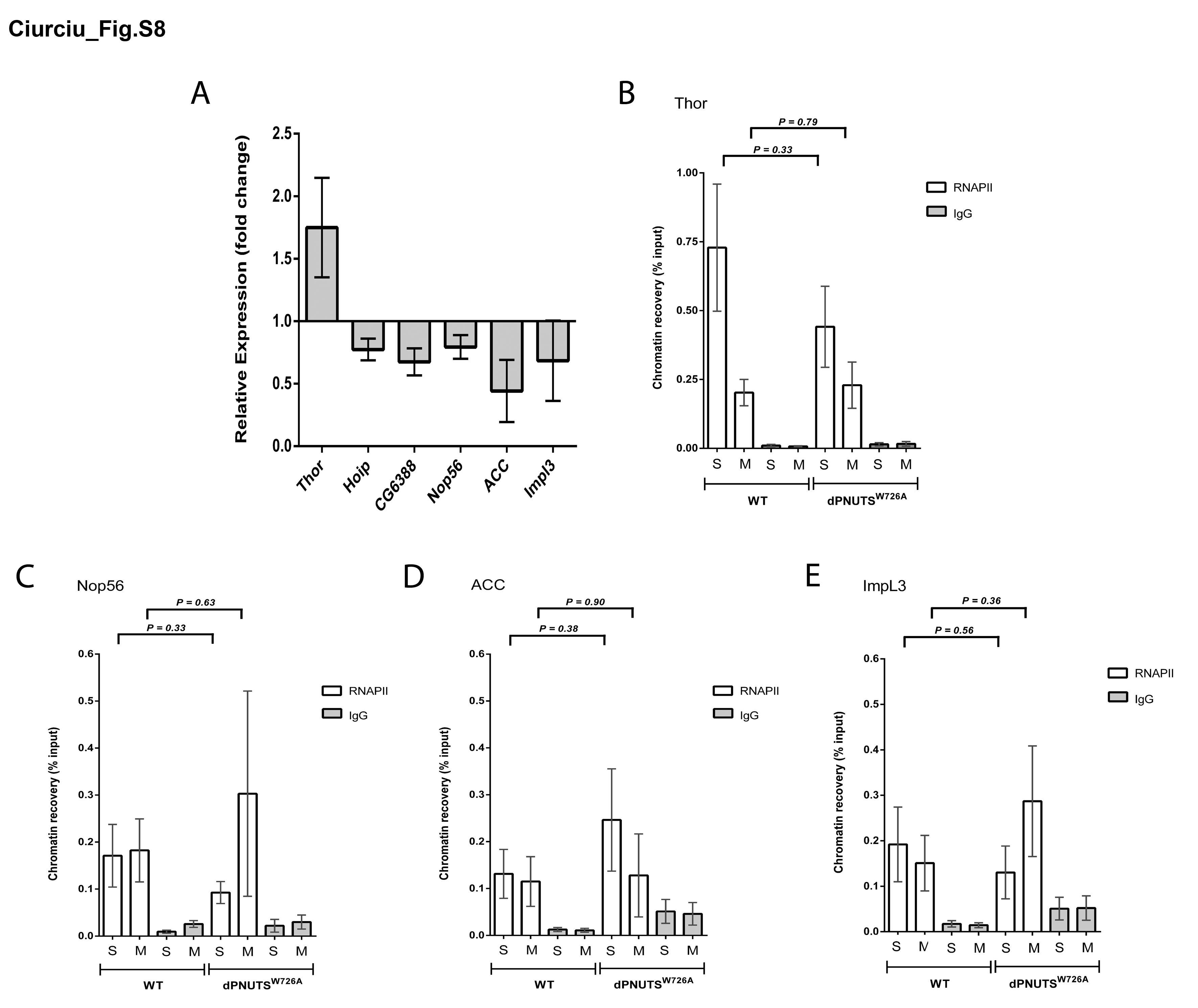

Supplement: Figure S8 — A) Expression levels of the indicated genes in larvae expressing dPNUTSW726A under the control of da-GAL4 relative to control larvae, as determined by qRT-PCR. Error bars represent the SE (n≥3 biological replicates). B–E) Chromatin immunoprecipitation (ChIP) analyses of the indicated genes from 3rd instar larval extracts using anti-total RNAPII (8WG16) antibody and mouse IgG antibody. Immunoprecipitated DNA was amplified by qPCR. The distribution at four loci (Thor, ImpL3, nop56 and ACC) was evaluated using primers positioned at the start (S) and middle (M), of the transcribed sequences. Percent input is the amount of precipitated DNA relative to input DNA. Error bars represent the SE of the mean (n≥3 biological replicates). (TIF) [file pgen.1003885.s008.tif]
