# Supplementary material for: PNUTS/PP1 Regulates RNAPII-Mediated Gene Expression and Is Necessary for Developmental Growth
Source: PLoS Genet. 2013 Oct 31;9(10):e1003885. doi: 10.1371/journal.pgen.1003885 (PMC3814315; doi:10.1371/journal.pgen.1003885)
Supplement: Table S1 — Rescue of dPNUTS mutant lethality by genomic transgene. Expected and observed genotype frequencies of adult progeny from complementation crosses with two independent insertions of a dPNUTS wild type trangene (n≥350 progeny/cross). (DOCX) [file pgen.1003885.s009.docx]

**Table S1.** Rescue of *dPNUTS* mutant lethality by genomic transgene.

| Cross  No. | Genotype | Expected (no rescue) % | Expected (complete rescue)  % | Observed % |
| --- | --- | --- | --- | --- |
| 1. | PNUTS13B/Cyo; P[PNUTS^+^ genomic, Line09]/TM6B X  PNUTS13B /Cyo; P[PNUTS^+^ genomic, Line09]/TM6B | | | |
|  | PNUTS^13B^ /PNUTS^13B^; P[PNUTS^+^ genomic, Line09]/TM6B | 0 | 22.2 | 18.6 |
|  | PNUTS^13B^ /PNUTS^13B^; P[PNUTS^+^ genomic, Line09]/ P[PNUTS^+^ genomic, Line09] | 0 | 11.1 | 11.8 |
|  | PNUTS^13B^ /Cyo; P[PNUTS^+^ genomic, Line09]/TM6B | 66.6 | 44.4 | 37.05 |
|  | PNUTS^13B^ /Cyo; P[PNUTS^+^ genomic, Line09]/ P[PNUTS^+^ genomic, Line09] | 33.3 | 22.2 | 32.4 |
| 2. | PNUTS^13B^ /Cyo; P[PNUTS^+^ genomic, Line03]/TM6B X  PNUTS^13B^ /Cyo; P[PNUTS^+^ genomic, Line03]/TM6B | | | |
|  | PNUTS^13B^ / PNUTS^13B^; P[PNUTS^+^ genomic, Line03]/TM6B | 0 | 22.2 | 19.2 |
|  | PNUTS^13B^ / PNUTS^13B^; P[PNUTS^+^ genomic, Line03]/ P[PNUTS^+^ genomic, Line03] | 0 | 11.1 | 12.8 |
|  | PNUTS^13B^ /Cyo; P[PNUTS^+^ genomic, Line03]/TM6B | 66.6 | 44.4 | 42.4 |
|  | PNUTS^13B^ /Cyo; P[PNUTS^+^ genomic, Line03]/ P[PNUTS^+^ genomic, Line03] | 33.3 | 22.2 | 25.6 |
