# Supplementary material for: PNUTS/PP1 Regulates RNAPII-Mediated Gene Expression and Is Necessary for Developmental Growth
Source: PLoS Genet. 2013 Oct 31;9(10):e1003885. doi: 10.1371/journal.pgen.1003885 (PMC3814315; doi:10.1371/journal.pgen.1003885)
Supplement: Table S5 — Comparison of RNA-Seq and qRT-PCR data, showing log2 fold change in expression of the indicated loci in dPNUTS mutants relative to control. (DOCX) [file pgen.1003885.s013.docx]

**Table S5**. Comparison of RNA-Seq and qRT-PCR data, showing log_2_ fold change in expression in *dPNUTS* mutants relative to control.

|  | **Gene name** | **dPNUTS[9B]/ dPNUTS[9B]** | | **dPNUTS[13B]/ dPNUTS[13B]** | |
| --- | --- | --- | --- | --- | --- |
|  |  | **RNA-seq** | **qRT-PCR** | **RNA-seq** | **qRT-PCR** |
| **UP** | Thor | 1.55 | 1.4 | 1.58 | 1.55 |
|  | Hid | 1.53 | 2.58 | 1.96 | 2.96 |
|  | p53 | 1.76 | 1.55 | 2.137 | 1.73 |
| **DOWN** | Hoip | 1.64 | 2.65 | 2.117 | 2.07 |
|  | CG4038 | 0.73 | 1.53 | 1.434 | 2.02 |
|  | CG6388 | 2.608 | 2.55 | 3.49 | 2.57 |
|  | CG6712 | 1.39 | 1.51 | 1.68 | 1.47 |
|  | nop56 | 1.415 | 1.87 | 1.32 | 1.68 |
|  | CG18600 | 1.36 | 1.92 | 1.717 | 1.76 |
|  | CG3756 | 1.44 | 0.68 | 1.31 | 0.59 |
|  | RPI135 | 0.55 | 1.09 | 1.08 | 0.96 |
|  | RPII215 | 0.08 | 0.14 | 0.014 | 0.32 |
|  | CG3523 | 1.217 | 0.34 | 1.68 | 0.66 |
|  | CG11198 | 1.18 | 1.12 | 1.49 | 1.59 |
|  | Tpi | 0.35 | 0.59 | 0.86 | 0.57 |
|  | GAPDH2 | 1 | 1.36 | 1.81 | 1.42 |
|  | ImpL3 | 1.45 | 2.04 | 1.21 | 2.02 |
