# Supplementary material for: PNUTS/PP1 Regulates RNAPII-Mediated Gene Expression and Is Necessary for Developmental Growth
Source: PLoS Genet. 2013 Oct 31;9(10):e1003885. doi: 10.1371/journal.pgen.1003885 (PMC3814315; doi:10.1371/journal.pgen.1003885)
Supplement: Text S1 — Additional information including detailed genotypes and primer sequences as well as methodology for supplementary figures. (DOCX) [file pgen.1003885.s014.docx]

**Text S1**

**List of genotypes**

Fig.2B-D

w^1118^; dPNUTS^exKG^/dPNUTS^exKG^

w^1118^; dPNUTS^9B^/CyO, twi-GFP

w^1118^; dPNUTS^9B^/dPNUTS^9B^

w^1118^; dPNUTS^13B^/CyO, twi-GFP

w^1118^; dPNUTS^13B^/dPNUTS^13B^

w^1118^; dPNUTS^KG^/CyO, twi-GFP

w^1118^; dPNUTS^KG^/dPNUTS^KG^

Fig.2E

w^1118^ (isogenic for autosomes)

GMR-hid, FRT40A, l(2)/CyO; ey-GAL4, UAS-FLP^1^

GMR-hid, FRT40A, l(2)/ dPNUTS^9B^, FRT40A; ey-GAL4, UAS-FLP^1^

GMR-hid, FRT40A, l(2)/ dPNUTS^13B^, FRT40A; ey-GAL4, UAS-FLP^1^

GMR-hid, FRT40A, l(2)/ dPNUTS^KG^, FRT40A; ey-GAL4, UAS-FLP^1^

GMR-hid, FRT40A, l(2)/ dPNUTS^exKG^, FRT40A; ey-GAL4, UAS-FLP^1^

Fig.3 and Fig.S3

hsFLP^122^; dPNUTS^13B^, FRT40A/ Ubi-GFPnls, FRT40A

hsFLP^122^; dPNUTS^13B^, FRT40A/ Ubi-GFPnls, FRT40A, M(RpL27A)

Fig. 5C

da-GAL4/+; UAS-HM-dPNUTS^WT^  line3/+

da-GAL4/+; UAS-HM-dPNUTS^W726A^  line1/+

Fig. 6A-C

AB1-GAL4, UAS-HM-dPNUTS^WT^  line3

AB1-GAL4, UAS-HM-dPNUTS^W726A^  line1

Fig. 6D

w^1118^

da-GAL4, UAS-HM-dPNUTS^WT^  line3

da-GAL4, UAS-HM-dPNUTS^W726A^  line1

Fig. 6E

GMR-hid, FRT40A, l(2)/ PNUTS^9B^, FRT40A; ey-GAL4, UAS-FLP^1^/+

GMR-hid, FRT40A, l(2)/ PNUTS^9B^, FRT40A; ey-GAL4, UAS-FLP^1^/ UAS-HM-dPNUTS^WT^ line3

GMR-hid, FRT40A, l(2)/ PNUTS^9B^, FRT40A; ey-GAL4, UAS-FLP^1^/ UAS-HM-dPNUTS^W726A^  line1

GMR-hid, FRT40A, l(2)/ PNUTS^13B^, FRT40A; ey-GAL4, UAS-FLP^1^/+

GMR-hid, FRT40A, l(2)/ PNUTS^13B^, FRT40A; ey-GAL4, UAS-FLP^1^/ UAS-HM-dPNUTS^WT^  line3

GMR-hid, FRT40A, l(2)/ PNUTS^13B^, FRT40A; ey-GAL4, UAS-FLP^1^/ UAS-HM-dPNUTS^W726A^  line1

Fig. 6F

GMR-hid, FRT40A, l(2)/ PNUTS ^KG572^, FRT40A; ey-GAL4, UAS-FLP^1^/+

GMR-hid, FRT40A, l(2)/ PNUTS^KG572^, FRT40A; ey-GAL4, UAS-FLP^1^/ PP187B^1^

GMR-hid, FRT40A, l(2)/ PNUTS^KG572^, FRT40A; ey-GAL4, UAS-FLP^1^/ PP187B^hs46^

Fig. 7B

w^1118^ (isogenic for autosomes)

w^1118^; dPNUTS^9B^/dPNUTS^9B^

w^1118^; dPNUTS^13B^/dPNUTS^13B^

w^1118^; dPNUTS^KG^/dPNUTS^KG^

w^1118^; dPNUTS^exKG^/dPNUTS^exKG^

Fig. 7C

w^1118^

da-GAL4, UAS-HM-dPNUTS^WT^  line3

da-GAL4, UAS-HM-dPNUTS^W726A^  line1

Fig. 7D,E

AB1-GAL4, UAS-H2B-YFP

AB1-GAL4, UAS-HM-dPNUTS^W726A^ line1

Fig.S2

AB1-GAL4, UAS-H2B-YFP

AB1-GAL4, UAS-PNUTS RNAi (NIG-FLY, 31657R-3)

Fig.S8

w^1118^

da-GAL4, UAS-HM-dPNUTS^W726A^ line1

**Supplementary Methods**

**Growth arrest experiment**

*PNUTS^9B^* and *PNUTS^13B^* were balanced with *Cyo, twi-GFP*. For each genotype, 10 females and 6 males were allowed to mate for 2 days and were then transferred to plates with laying apple juice medium at 25°C. The first-day egg collections were discarded, and, starting on the second day, a 4 hr egg collection regimen was established. After 24 hr, 30 homozygous (GFP negative) and 30 heterozygous (GFP positive) eggs from each genotype were transferred to separate fresh agar plates. After each 24 hr, the number of living larvae was counted and their size was compared until all larvae died.

**Imaging of adult eyes**

Flies were collected in micro-centrifuge tubes and frozen and stored at -20°C until imaging. Flies were imaged using a Leica MZ10F stereomicroscope (Leica).

***dPNUTS* cDNA isolation and sequence analysis**

5 x 10^6^ *Drosophila* 3rd instar larval cDNAs were screened using PP1β9C as bait in the two-hybrid assay as [1, 2]. cDNAs containing the complete dPNUTS open reading frame were obtained by hybridization using the screening procedure described in [3]. In brief, 1 x 10^6^ clones from a 3^rd^ instar larval library [4] were screened in pools by Southern Blotting and PCR, using the longest two-hybrid clone (DB388: nucleotides 1894-4831 of dPNUTS or a PCR product from the 3’ end of DB388 (nucleotides 3239-4096 of dPNUTS) as probes. These approaches identified full-length clones of dPNUTS-S and dPNUTS, respectively, representing the two alternative mRNA splice variants of the d*PNUTS* gene. Identification of the intron and exon structures of PNUTS and dPNUTS was done by comparison of the following sequences: d*PNUTS* cDNA reported in this study; AE003588, d*PNUTS* genomic; AJ544537, human *PNUTS* mRNA; AB088097, human *PNUTS* genomic.

**Yeast two-hybrid assays**

GAL4 activation domain-dPNUTS fusion constructs were made as follows: A *Bam*HI site was introduced immediately 5’ of the translation start and a *Sal*I site at the 3’ end of both *dPNUTS* and *dPNUTS-S* by PCR using the following primers: (+) 5’-CTAAG­GATCCAAATGCCTCGTATAGTTCC-3’,(-)5’-GGGGTCGACAGACCGGAATTCGGC­GG-3’. The whole open reading frames of *dPNUTS* and *dPNUTS-S* were then introduced as *Bam*HI/*Sal*I fragments into the *Bam*HI/*Xho*I sites of pACT. For the 2-hybrid assays: GAL4 DNA-binding domain-PP1c fusion constructs pAS2-PP1α87B, pAS2-PP1α13C, pAS2-PP1α96A and pAS2-PP1β9C have been previously described [2, 5]. Y190 yeast cells were transformed with pAS2 and pACT constructs and transformed cells were selected on drop-out base with agar plates (BIO 101) lacking leucine, tryptophan and histidine. Colonies appearing after 4-5 days of incubation at 30^o^C were tested for β-galactosidase activity using a filter lift assay described by Clontech.

**dPNUTS and PP1 antisera**

Guinea pig anti-PNUTS and rabbit anti-PP1 antibodies were generated by Moravian-Biotechnology and Eurogentec respectively using the following peptides as antigens: KLEVDNVPDHPNGNL (residues 789-792 of dPNUTS); KLFSILFHSPRTLVA (residues 581-594 of dPNUTS-S); RGARPGKNVQLSEGE and SDPDKDTMGWGENDR (residues 18-32 and 205-219, respectively of PP187B).

**Analysis of RNAPII CTD phosphorylation levels by Western Blotting**

The following monoclonal antibodies directed against phospho-CTD marks were obtained from Chromotek: 3E10 (Ser2-P); 6D7 (Thr4-P); 3E8 (Ser5-P); 4E12 (Ser7-P). For relative quantitation, signals were captured using an ImageQuant biomolecular imager (GE Healthcare) and quantified using ImageJ (<http://rsbweb.nih.gov/ij/>).

**Co-immunoprecipitation from *Drosophila* extracts**

Nuclear extracts, prepared from approximately 200 µl of dechorionated embryos of each genotype, were incubated with primary antibodies overnight and then another 2-4 hrs the next day with either GammaBind Plus Sepharose (Amersham) or protein A-coated magnetic beads (Millipore).

**Co-immunoprecipitation from S2 cell extracts**

2 x 10^6^ S2R+ cells in 2ml Schneider’s were seeded out 2-3 hours before transfection. Cells were either transfected or co-transfected with constructs containing Flag-Myc-dWdr82 or GFP-dPNUTS-Myc using Effectene transfection reagent (Qiagen) according to the manufacturer’s protocol for the transient transfection of adherent cells. After 48-72 hrs incubation, cells were harvested at 4000 rpm for 3 mins. Immunoprecipitation was carried out using magnetic GFP-Trap beads (Chromotek) according to the manufacturer’s protocol. Anti-Myc antibody (A14, 1:1000) was used for immunoblotting of S2 cell extracts.

**Chromatin Immunoprecipitation (ChIP) from larval extracts**

ChIP experiments were performed from wandering 3rd instar larvae, as described previously [7]. For the immunoprecipitations, 25 µg of chromatin was incubated overnight with antibody and another 4 hrs the next day with protein A or G coated magnetic beads (Diagenode or Millipore). The antibodies used in the IP were: mouse anti-total RNAPII (8WG16, Covance) and mouse IgG. A minimum of 3 biological replicates was done for each genotype. For the qPCR analysis, reactions were done in duplicates and the quantity of DNA bound by specific antibodies was calculated by % Input. Primers used for PCR are given in the table below.

**Immunostaining of wing discs and whole mount salivary glands**

Tissues were dissected from 3^rd^ instar larvae were fixed in 3.7% paraformaldehyde in 1x phosphate-buffered saline (PBS) for 20 minutes. Tissues were washed in 1x PBS for 15 minutes and then blocked in 1x PBS, 5% bovine serum albumin (BSA), 0.1% Triton X-100 (blocking solution) for 30 minutes at room temperature or a minimum of 2 hours at 4°C. Tissues were incubated with primary antibody in blocking solution overnight at 4°C. Tissues were washed in PBST (1x PBS, 0.1% Triton X-100) and incubated with secondary antibody conjugated to Alexa-Fluor 488, 555 or 633 (1:500, Molecular Probes) for 2 hours at room temperature in the dark. Tissues were washed two times in PBST for 10 minutes each time followed by incubation with TO-PRO-3 (Invitrogen, 1:1000 in 1x PBS) for visualising DNA. Tissues were mounted in Vectashield mounting medium (Vector Laboratories).

**Immunostaining of polytene chromosomes**

Polytene chromosome squashes were done as described previously [8]. In brief, salivary glands of wandering larvae were fixed with 3.7% paraformaldehyde dissolved in phosphate-buffered saline (PBS) and then incubated in 45% acetic acid for 1 minute. Slides were blocked in PBST (PBS+ 0.1% Tween-20) + 5% BSA for 1 hour at 25°C and incubated overnight at 4°C with primary antibodies. Slides were washed in PBST and incubated with secondary antibodies for 1 hour at 25°C: Alexa-Fluor 488-conjugated goat anti-mouse IgG, Alexa-Fluor 488-conjugated goat anti-guinea pig IgG and Alexa-Fluor 555-conjugated goat anti-rabbit IgG (Molecular Probes), were used at 1:500 dilutions. For DNA staining, slides were incubated with DAPI or TO-PRO-3 in PBST for 10 minutes at 25°C, washed again and covered with Vectashield.

**Expression Analysis Systematic Explorer (EASE) analysis**

Statistical measurement of GO term enrichment comparing genes differentially expressed to genes expressed in developmentally matched *w^1118^* controls was determined using an EASE score (P< 0.05) [9].

**Ingenuity IPA analysis**

Data sets containing identifiers for the human orthologues of differentially expressed (DE) *Drosophila* genes were uploaded into Ingenuity IPA. Each identifier was mapped to its corresponding object in the Ingenuity Knowledge Base. Network Eligible molecules, were overlaid onto a global molecular network developed from information contained in the Ingenuity Knowledge Base. Networks were algorithmically generated from Network Eligible Molecules based on their connectivity. All edges are supported by at least one reference from the literature, from a textbook, or from canonical information stored in the Ingenuity Knowledge Base. The generated networks were used by IPA to automatically predict ‘upstream regulators’ of DE genes. The term “upstream regulator” refers to any molecule that can affect the expression of another molecule.  Upstream regulators cover the gamut of molecule types founds in the literature, from transcription factors, to cytokines, microRNAs, receptors, kinases, chemicals and drugs. Upstream Regulator Analysis is based on expected causal effects between Upstream regulators and targets; the expected causal effects are derived from the literature compiled in the Ingenuity Knowledge Base. The analysis examines the known targets of each upstream regulator in the dataset, compares the targets’ actual direction of change to expectations derived from the literature, then issues a prediction for each upstream regulator. IPA uses a z-score algorithm to make predictions. The z-score algorithm is designed to reduce the chance that random data will generate significant predictions.

**Oligonucleotide primer sequences**

| **Name** | **Sequence (5’-3’)** | **Purpose** |
| --- | --- | --- |
| PNUTSATGex+ | ACGCAGGAGTTTTCGGAGAG | Mutant characterisation |
| PNUTSATGex- | ATTGTGGAGACCCTCGGTGA | Mutant characterisation |
| PNUTS3ex+ | GCTGCCCGAGTGTGAGTCTA | Mutant characterisation |
| PNUTS3ex- | GTTCGGTTTAATGGCAAACGTAATG | Mutant characterisation |
| PNUTS Fw | GCCAAGATCGACATCAACAA | qRT-PCR |
| PNUTS Rev | CTTGCGCTTCACCACCTT | qRT-PCR |
| dPNUTS-S Fw | GGATCGAGTTTGAACTGCAAG | qRT-PCR |
| dPNUTS-S Rev | AACATCAGGCAGCGACAAC | qRT-PCR |
| Thor Fw | AACCCTCTACTCCACCACTCC | qRT-PCR |
| Thor Rev | ACTTGCGGAAGGGAGTACG | qRT-PCR |
| Hid Fw | GGCCGTAAAGTTGTCGTAGC | qRT-PCR |
| Hid Rev | GACCTCCACGCCGTTATC | qRT-PCR |
| RPI135 Fw | ACCGTGCGGACTGTTAAATC | qRT-PCR |
| RPI135 Rev | GACCCAAGTGTTTGCCATCT | qRT-PCR |
| Nop56 Fw | CAACTGATCCAGCAAAGCAA | qRT-PCR |
| Nop56 Rev | TCCAGTGCAGTCACTTTGGA | qRT-PCR |
| Hoip Fw | CCATCGAGATTCTGCTCCAT | qRT-PCR |
| Hoip Rev | GTGATCTGCGACTTGAGCTG | qRT-PCR |
| CG6712 Fw | CGACGACAAGAAGACACGAA | qRT-PCR |
| CG6712 Rev | AGATTGGCCACCTCTGTTTG | qRT-PCR |
| CG4038 Fw | GAGGAGGACGAGGATTTGGT | qRT-PCR |
| CG4038 Rev | GGTCGTTTTGGCAGGAGTAA | qRT-PCR |
| CG6388 Fw | ATCCGGATATTTGTGCGTGT | qRT-PCR |
| CG6388 Rev | CTTCAGCTGCTGGGGATTAC | qRT-PCR |
| CG18600 Fw | GGCCAAACATGACTTGAGGT | qRT-PCR |
| CG18600 Rev | ACAAGGAGGCAACCAAAGTG | qRT-PCR |
| CG3756 Fw | GAGGAGTATCGGGTGAAGCA | qRT-PCR |
| CG3756 Rev | ATTTGCAATGGCGGGATAC | qRT-PCR |
| Tpi FW | CTTGGAGATGTTGTCGCTCA | qRT-PCR |
| Tpi Rev | CCTACGCCCAGAAGATCAAG | qRT-PCR |
| GAPDH2 Fw | GGTGATCAACGACAACTTCG | qRT-PCR |
| GAPDH2 Rev | CCAGTGGAAGCTGGAATGAT | qRT-PCR |
| ImpL3 Fw | CTTGACCACGGATGTCACAC | qRT-PCR |
| ImpL3 Rev | GATACACCTCCTGGGCCATT | qRT-PCR |
| CG3523 Fw | CCACCATCGAGGAGTTCAAG | qRT-PCR |
| CG3523 Rev | CACCGAAGAACTGTTGGTCA | qRT-PCR |
| CG11198 Fw | TACGAGAGCCAGTCGAGGAT | qRT-PCR |
| CG11198 Rev | GGCTATGCTGCGCTTAACA | qRT-PCR |
| 18S Fw | CGCAAGATCGTTATATTGGTTG | qRT-PCR |
| 18S Rev | GCTGCCTTCCTTAGATGTGG | qRT-PCR |
| RpII215 Fw | ACCAGCTAGGCGACATTCC | qRT-PCR |
| RpII 215 Rev | GATCGACACCGAGCATGAC | qRT-PCR |
| p53 Fw | GGACTTGCGCTTCTTGCTAT | qRT-PCR |
| p53 Rev | TGTATCGGGCGAAAAGAAAC | qRT-PCR |
| Thor-S Fw | CATAGCAGCCACACAAGCTC | qPCR (from ChIP) |
| Thor-S Rev | GGTGAAGCGGACATCTTAGC | qPCR (from ChIP) |
| Thor-M Fw | TTATCTACGAGCGGGCTTTC | qPCR (from ChIP) |
| Thor-M Rev | ACTTGCGGAAGGGAGTACG | qPCR (from ChIP) |
| ImpL3-S Fw | GGGCCAACAGACTGTCCTTA | qPCR (from ChIP) |
| ImpL3-S Rev | AATCATAGGCACGTGATAGCAA | qPCR (from ChIP) |
| ImpL3-M Fw | ATCACCTCGTAGGCGGAGT | qPCR (from ChIP) |
| ImpL3-M Rev | CGTTTGGTCTGGAGTGAACA | qPCR (from ChIP) |
| nop56-S Fw | TGCCGAATATATGCCGATTT | qPCR (from ChIP) |
| nop56-S Rev | GGCTTGCTATGGTCACACTTG | qPCR (from ChIP) |
| nop56-M Fw | CGTATTGGAGCGGGTCTTTA | qPCR (from ChIP) |
| nop56-M Rev | GCACCCAATCTGCAGTCTTT | qPCR (from ChIP) |
| CAA-S Fw | AGCTCGCTTTACCACTCTGC | qPCR (from ChIP) |
| CAA-S Rev | CATCTACAAATCGTGCGGAAC | qPCR (from ChIP) |
| CAA-M Fw | CCACGCATTTGTCGTAGTGT | qPCR (from ChIP) |
| CAA-M Rev | CAGCGCTATAGGAACGGAAT | qPCR (from ChIP) |

**Supplementary References**

1. Bennett, D. and L. Alphey, *PP1 binds Sara and negatively regulates Dpp signaling in Drosophila melanogaster.* Nat. Genet., 2002. **31**(4): p. 419-423.

2. Bennett, D., B. Szöor, and L. Alphey, *The chaperone-like properties of mammalian Inhibitor-2 are conserved in a Drosophila homologue.* Biochemistry, 1999. **38**(49): p. 16276-16282.

3. Alphey, L., *PCR-based method for isolation of full-length clones and splice variants from cDNA libraries.* BioTech., 1997. **22**: p. 481-486.

4. Brown, N. and F. Kafatos, *Functional cDNA libraries from Drosophila embryos.* J. Mol. Biol., 1988. **203**: p. 425-437.

5. Alphey, L., et al., *KLP38B: a mitotic kinesin-related protein that binds PP1.* J. Cell Biol., 1997. **138**(2): p. 395-409.

6. Stock, J.K., et al., *Ring1-mediated ubiquitination of H2A restrains poised RNA polymerase II at bivalent genes in mouse ES cells.* Nat Cell Biol, 2007. **9**(12): p. 1428-35.

7. Zsindely, N., et al., *The loss of histone H3 lysine 9 acetylation due to dSAGA-specific dAda2b mutation influences the expression of only a small subset of genes.* Nucleic Acids Res, 2009. **37**(20): p. 6665-80.

8. Ciurciu, A., et al., *The Drosophila histone acetyltransferase Gcn5 and transcriptional adaptor Ada2a are involved in nucleosomal histone H4 acetylation.* Mol Cell Biol, 2006. **26**(24): p. 9413-23.

9. Hosack, D.A., et al., *Identifying biological themes within lists of genes with EASE.* Genome Biol, 2003. **4**(10): p. R70.
